# Emerging Epigenetic‐Based Nanotechnology for Cancer Therapy: Modulating the Tumor Microenvironment

**DOI:** 10.1002/advs.202206169

**Published:** 2023-01-04

**Authors:** Jiaxin Zhang, Leaf Huang, Guangbo Ge, Kaili Hu

**Affiliations:** ^1^ Shanghai Frontiers Science Center of TCM Chemical Biology Institute of Interdisciplinary Integrative Medicine Research Shanghai University of Traditional Chinese Medicine Shanghai 201203 China; ^2^ Division of Pharmacoengineering and Molecular Pharmaceutics Eshelman School of Pharmacy University of North Carolina at Chapel Hill Chapel Hill NC 27599 USA

**Keywords:** cancer treatment, DNA methylation, histone modification, nanotechnology, tumor microenvironment

## Abstract

Dysregulated epigenetic modifications dynamically drive the abnormal transcription process to affect the tumor microenvironment; thus, promoting cancer progression, drug resistance, and metastasis. Nowadays, therapies targeting epigenetic dysregulation of tumor cells and immune cells in the tumor microenvironment appear to be promising adjuncts to other cancer therapies. However, the clinical results of combination therapies containing epigenetic agents are disappointing due to systemic toxicities and limited curative effects. Here, the role of epigenetic processes, including DNA methylation, post‐translational modification of histones, and noncoding RNAs is discussed, followed by detailed descriptions of epigenetic regulation of the tumor microenvironment, as well as the application of epigenetic modulators in antitumor therapy, with an emphasis on the epigenetic‐based advanced drug delivery system in targeting the tumor microenvironment.

## Graphical abstract

The epigenetic‐based nanotechnology is a particularly attractive strategy for accurate tumor delivery of one or several different anticancer agents as well as for therapeutic resistance reversal and tumor microenvironment modulation. This review summarizes the emerging application of epigenetic‐based drug delivery systems in targeting the tumor microenvironment to promote the translation of epigenetic‐based nanotechnology for cancer therapy.

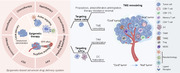

## 1 Introduction

Epigenetic regulation has emerged as a potential means of cancer intervention.^[^ ^1^ ^]^ Epigenetics is a reversible and dynamic process that regulates gene expression without changing the DNA sequence. Nucleosomes, the basic functional unit of chromatin, contain a histone octamer core (H2A, H2B, H3, and H4) wrapped by a recurrent 147 bp stretch of DNA.^[^ ^2^ ^]^ Covalent histone modification and DNA methylation cooperatively regulate chromatin structure and gene expression.^[^ ^3^ ^]^ Enzymes that regulate epigenetic modification have been classified into writers, erasers, and readers. The enzymes adding specific marks to DNA or histones are termed a “writers,” including DNA methyltransferase (DNMT), histone acetyltransferase (HAT), histone lysine methyltransferase (KMT), and protein arginine methyltransferase (PRMT),^[^ ^4^ ^]^ while the enzymes removing the posttranslational modification are termed “erasers,” including histone deacetylase (HDAC) and histone lysine demethylase (KDM).^[^ ^1^ ^]^ Furthermore, bromodomain and chromodomain proteins are known as “readers” that recognize acetyl or methyl groups, respectively.^[^ ^1^ ^]^ Alterations in DNA methylation and histone modification regulate the accessibility and function of chromatin in tumor progression. Epigenetic therapy has been developed to regulate DNA methylation and histone modification that facilitate malignancy progression.^[^ ^1^ ^]^

Innate and adaptive immunity cooperate against progressive tumors. Genes related to regulating immune cells may be epigenetically modified in cancer; thus, specially affecting the progression of cancer. Epigenetic reprogramming of immune cells mediates tumor immunity and affects the progression of cancer. Epigenetic modifications may regulate the transition between “cold” tumors (immune repressive phenotype) and “hot” tumors (immune permissive phenotype) by affecting the function and phenotype of immune cells in the tumor microenvironment (TME).^[^ ^5^ ^]^ The TME, a complex environment that promotes tumor progression, metastasis, and recurrence, consists of stromal cells, immune cells, tumor cells, extracellular matrix (ECM), secretory molecules, and blood and lymphatic vascular networks. Immune cells include T and B lymphocytes, dendritic cells (DCs), myeloid‐derived suppressor cells (MDSCs), neutrophils, tumor‐associated macrophages (TAMs), natural killer (NK) cells, and so on.^[^ ^6^ ^]^ The stromal cells are composed of cancer‐associated fibroblasts (CAFs), mesenchymal stromal cells, and pericytes.^[^ ^6^ ^]^ The suppression of the immune system is vitally interrelated with the poor prognosis of cancer. Recent studies have extensively focused on the epigenetic regulation of tumor cells, immune cells, and stromal cells in the TME.^[^ ^7^ ^]^

Epigenetic therapy can further synergize with other anticancer therapies (including immunotherapy, chemotherapy, radiotherapy, molecularly targeted therapy, photothermal therapy, photodynamic therapy, photoacoustic imaging, and other therapies) to improve antitumor efficiency through different mechanisms. Furthermore, the combination of epigenetic modulators and other anticancer agents can overcome drug resistance in tumor treatment.^[^ ^8^ ^]^ The limited bioavailability of epigenetic modulators and other anticancer agents is a huge challenge, but the development of novel nano‐targeting technology may greatly improve the therapeutic efficiency of these drugs.

**Scheme 1:**
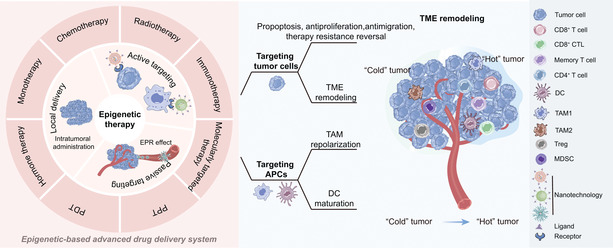
Epigenetic modulator‐based nanotechnology can effectively transfer “cold” tumors into “hot” tumors by targeting tumor cells and APCs (e.g., macrophages and DCs); and then, improve antitumor therapy.

Here, epigenetic regulation in the TME and the application of epigenetic‐based nanotechnology for cancer therapy are summarized. A profound understanding of the regulatory effect of epigenetic alteration on tumor cells and immune cells will provide effective strategies to improve the tumor immune microenvironment. In this review, the current nano‐therapeutic strategies of epigenetic therapy as well as their synergy with other therapeutic methods are reviewed and discussed (**Scheme** **1**).

## 2 Effect of Epigenetic Regulation on Tumor Therapy

### 2.1 Epigenetic Modifications

#### 2.1.1 DNA Methylation

**Figure 1:**
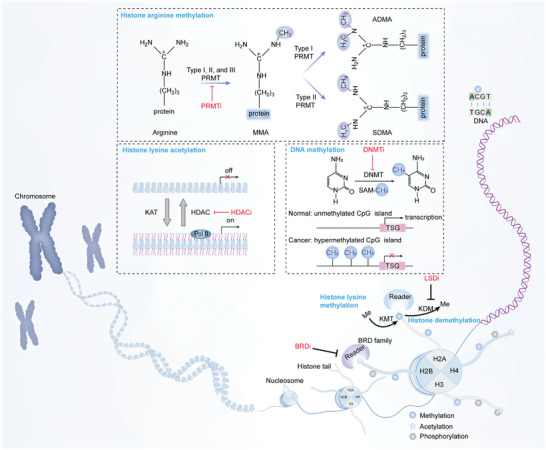
The mechanisms of DNA methylation and histone modification. The basic unit of chromatin is the nucleosome in which DNA is tightly wrapped around a histone octamer core (consisting of H2A, H2B, H3, and H4). DNA methylation: DNA methyl transferases (DNMTs) can transfer the methyl of the S‐adenosylmethionine (SAM) donor to DNA at position 5 in cytosine. In normal cells, CpG islands at tumor suppressor gene (TSG) promoters are usually unmethylated and are characteristic of active transcription genes; however, abnormal DNA hypermethylation is related to tumorigenesis and transcriptional inactivation. Histone acetylation: histone lysine acetyltransferases (KATs) catalyze the acetylation of histone; thus, promoting chromatin opening and active gene transcription, while histone deacetylases (HDACs) initiate the process of histone deacetylation, which is less accessible by forming a condensed state. Acetylation of lysine residues can be read by bromodomain‐containing protein (BRD) family. Histone lysine methylation: histone lysine methyltransferases (KMTs) methylate lysine residues, which can be erased by histone lysine demethylases (KDMs). Histone arginine methylation: three types of protein arginine methyltransferases (PRMTs) can catalyze histone arginine methylation. Several epigenetic drugs including DNMT inhibitor (DNMTi), HDAC inhibitor (HDACi), BRD inhibitor (BRDi), lysine‐specific demethylase inhibitor (LSDi), and PRMT inhibitor (PRMTi), which act on DNA methylation, histone deacetylation, histone demethylation, and histone arginine methylation, have been approved for clinical cancer treatment.

DNA methylation is the most widely known epigenetic mechanism of gene regulation (**Figure** **1**). In eukaryotes, DNA methylation is thought to be a chemical modification process in which the cytosine of cytosine–phosphate–guanine (CpG) dinucleotides can be converted into 5‐methylcytosine under the catalysis of DNMTs responsible for catalytically transferring the methyl of the S‐adenosylmethionine (SAM) donor.^[^ ^9^ ^]^ In the DNMT family, DNMT1 appears to maintain DNA methylation during DNA replication, whereas DNMT3A and DNMT3B catalyze the de novo methylation of DNA.^[^ ^10^ ^]^ CpG dinucleotide‐rich sites are referred to as CpG islands. In normal cells, CpG islands at gene promoters are usually unmethylated; however, abnormal hypermethylation leads to transcriptional inactivation.^[^ ^11^ ^]^ Cancer is characterized by genome‐wide hypomethylation and site‐specific hypermethylation, and tumor mutations appear in methylated CpG sites.^[^ ^12^ ^]^ Active DNA demethylation is positively regulated by oxidation catalysis of 5‐methylcytosine, which is catalytically oxidized by the ten‐eleven translocation (TET) of *α*‐ketoglutarate (*α*‐KG)‐dependent dioxygenase.^[^ ^10^, ^13^ ^]^

DNA methylation inhibitors characterized by nucleoside analogs with a modified cytosine ring can be incorporated into newly synthesized DNA or RNA and then covalently bind with DNMTs, resulting in hypomethylation of newly synthesized DNA.^[^ ^14^ ^]^ Small molecule inhibitors of DNMT (DNMTi), also known as hypomethylation agents, are the most common epigenetic therapy for cancer. The DNA methylation inhibitors 5‐azacitidine (5‐Aza)^[^ ^15^ ^]^ and 5‐Aza‐2′‐deoxycytidine (decitabine)^[^ ^16^ ^]^ have also been approved for myelodysplastic syndrome therapy (**Table** **1**). 5‐Aza can be incorporated into DNA and RNA, while decitabine is only inserted into DNA.^[^ ^16^ ^]^ In addition, some non‐nucleoside analogs including RG108,^[^ ^17^ ^]^ (‐)‐epigallocatechin‐3‐gallate,^[^ ^18^ ^]^ hydralazine, and procainamide^[^ ^19^ ^]^ have been reported to directly target the catalytic site instead of being incorporated into DNA to prevent abnormal hypermethylation of DNA.^[^ ^14^ ^]^

**Table 1:** Epigenetic drugs for solid tumors that are in clinical trials (clinicaltrials.gov)

Category	Mechanism of the epigenetic regulators	Epigenetic regulators	Drug/inhibitors	Trial iD	Clinical trial status	Cancer type
Writers	Promotes DNA methylation	DNMT1, 3A, and 3B	Azacitidine (Vidaza)	NCT02940483	Early phase 1 /completed	Recurrent posterior fossa ependymoma
				NCT04891068	Phase 2/recruiting	Breast cancer
				NCT00384839	Phase 2/completed	Prostate cancer
				NCT01845805	Phase 2/completed	Pancreatic cancer
				NCT02009436	Phase 1/completed	NSCLC
				NCT02223052	Phase 1/completed	Solid tumor
				NCT00004062	Phase 1/completed	Thyroid cancer
				NCT02269943	Phase 2/ completed	Nasopharyngeal carcinoma
			Decitabine (5‐aza‐2'‐Deoxycytidine, Dacogen)	NCT00030615	Phase 1/completed	Advanced solid tumors
				NCT02316028	Phase 1/phase 2/completed	Colorectal cancer liver metastasis
				NCT00002980	Phase 1/completed	Melanoma or other advanced cancer
				NCT00477386	Phase 1/phase 2/completed	Ovarian cancer
				NCT00019825	Phase 1/completed	Esophageal cancer, lung cancer, malignant mesothelioma, and metastatic cancer
			Guadecitabine (SGI‐110)	NCT02429466	Phase 1/completed	Germ cell tumor and testis cancer
				NCT03075826	Phase 2/completed	Myeloproliferative neoplasms
				NCT01752933	Phase 2/completed	Hepatocellular carcinoma
			5‐Fluoro‐2‐Deoxycytidine	NCT00359606	Phase 1/completed	Neoplasms
		DNMT1	5‐aza‐4'‐Thio‐2'‐Deoxycytidine	NCT03366116	Phase 1/recruiting	Advanced solid tumors
	Catalyzes H3K27 methylation	EZH2	CPI‐1205	NCT02395601	Phase 1/completed	B‐cell lymphoma
			Tazemetostat (Tazverik)	NCT05567679	Early phase 1/not yet recruiting	Prostate cancer
				NCT05023655	Phase 2/recruiting	ARID1A mutated malignancies
				NCT04917042	Phase 2/recruiting	Peripheral nerve sheath tumors
				NCT02601937	Phase 1/completed	INI1‐negative tumors or synovial
				NCT04241835	Phase 1/recruiting	Hepatic impairment and advanced malignant solid tumor
				NCT02601950	Phase 2/active, not recruiting	INI1‐negative tumors and synovial sarcoma
				NCT03213665	Phase 2/active, not recruiting	Advanced solid tumors with EZH2, SMARCB1, or SMARCA4 gene mutations
				NCT03348631	Phase 2/active, not recruiting	Ovarian or endometrial cancer
				NCT02860286	Phase 2/completed	BAP1‐deficient mesothelioma
	Promotes H3K79 methylation	DOT1L	EPZ‐5676	NA	NA	NA
	Catalyzes histone arginine methylation	Type I PRMT	GSK3368715	NA	NA	NA
			MS023	NA	NA	NA
		PRMT3	SGC707	NA	NA	NA
		PRMT4	GSK3359088	NA	NA	NA
		PRMT5	GSK3326595	NCT04676516	Phase 2/completed	Breast cancer
			TNG908	NCT05275478	Phase 1/phase 2/recruiting	MTAP‐deleted solid tumors
			SH3765	NCT05015309	Phase 1/not yet recruiting	Advanced malignant tumor
		PRMT6	EPZ020411	NA	NA	NA
Erasers	Promotes histone deacetylation	HDAC class I, class II, and class IV	Vorinostat (Zolinza)	NCT00330161	Phase 2/completed	Prostate cancer
				NCT00907738	Phase 2/completed	Advanced cancer
				NCT00127127	Phase 1/completed	Solid tumors
				NCT00132067	Phase 2/completed	Primary peritoneal cavity cancer or recurrent ovarian epithelial cancer
				NCT00278395	Phase 2/completed	Renal cell cancer
				NCT00005634	Phase 1/completed	Advanced solid tumors
				NCT00788112	Phase 1/completed	Breast cancer
				NCT00134043	Phase 2/completed	Thyroid cancer
				NCT00128102	Phase 3/completed	Advanced malignant pleural mesothelioma
				NCT01175980	Phase 2/completed	Adenoid cystic carcinoma
				NCT00238303	Phase 2/completed	Glioblastoma multiforme
				NCT00121225	Phase 2/completed	Melanoma
			Belinostat	NCT01583777	Phase 1/completed	Advanced cancer
				NCT00413075	Phase 1/completed	Advanced solid tumor
				NCT00321594	Phase 1/phase 2/completed	Liver cancer
				NCT00301756	Phase 2/completed	Ovarian epithelial cancer, primary peritoneal cancer, or fallopian tube cancer
				NCT00589290	Phase 2/completed	Thymic carcinoma
			Panobinostat (Farydak, LBH589)	NCT00690677	Phase 2/completed	Colorectal cancer
				NCT00739414	Phase 1/completed	Advanced solid tumors
				NCT01007968	Phase 1/completed	Advanced solid tumors
				NCT00997399	Phase 1/completed	Advanced solid tumors
				NCT01013597	Phase 2/completed	Thyroid carcinoma
				NCT01222936	Phase 2/completed	Small cell lung cancer
				NCT00550277	Phase 2/completed	Renal cell carcinoma
				NCT01065467	Phase 1/completed	Melanoma
		HDAC class I	Romidepsin (depsipeptide, FK228)	NCT00106418	Phase 2/completed	Prostate cancer
				NCT00077337	Phase 2/completed	Colorectal cancer
				NCT00098813	Phase 2/completed	Thyroid cancer
				NCT00020202	Phase 2/completed	Small cell lung cancer or NSCLC
				NCT00106613	Phase 2/completed	Renal cell carcinoma
				NCT00085540	Phase 1/phase 2/completed	Gliomas
				NCT00084682	Phase 2/completed	Squamous cell carcinoma of the head and neck
			Entinostat (MS‐275)	NCT02897778	Phase 1/completed	Advanced solid tumors
				NCT00020579	Phase 1/completed	Advanced solid tumors
		HDAC class I and class IV	Mocetinostat (MGCD0103)	NCT00323934	Phase 1/completed	Advanced solid tumors
				NCT02236195	Phase 2/completed	Urothelial carcinoma
		HDAC 1, 3, and 6	Resminostat	NA	NA	NA
		HDAC1, 2, 3, and 10	Chidamide (Tucidinostat)	NCT05276713	NA/recruiting	HR^+^ breast cancer
				NCT02883374	Phase 2/unknown	Adenocystic carcinoma
		HDAC6	Ricolinostat	NA	NA	NA
			KA2507	NCT03008018	Phase 1/completed	Solid tumor
			Citarinostat	NA	NA	NA
	Mutations in isocitrate dehydrogenase (IDH), leading to DNA and histone hypermethylation	IDH1	Lvosidenib (AG‐120)	NCT04195555	Phase 2/recruiting	IDH1 mutations advanced solid tumors
				NCT02073994	Phase 1/active, not recruiting	Advanced solid tumors, including glioma, cholangiocarcinoma, and chondrosarcoma,with an IDH1 mutation
				NCT04278781	Phase 2/recruiting	IDH1 mutant chondrosarcoma
				NCT02989857	Phase 3/completed	IDH1 mutant cholangiocarcinoma
		IDH2	Enasidenib	NCT02273739	Phase 1/phase 2/completed	Solid tumor, glioma, intrahepatic cholangiocarcinoma, or chondrosarcoma with IDH2 mutation
	Demethylates mono‐ or di‐methylated lysines	LSD1	GSK2879552	NCT02034123	Phase 1/terminated	Small cell lung carcinoma
			Seclidemstat	NCT03895684	Phase 1/completed	Advanced solid tumors
			ORY‐1001	NA	NA	NA
Readers	Reads acetyl groups on histone lysines	BET	Molibresib (GSK525762)	NCT01587703	Phase 1/completed	Carcinoma
			Birabresib (OTX015)	NCT02259114	Phase 1/completed	Advanced solid tumors
			BAY1238097	NA	NA	NA
			CPI‐0610	NA	NA	NA
		BRD4	JQ1	NA	NA	NA

#### 2.1.2 Histone Modifications

Histones are involved in a large number of post‐translational modifications, including acetylation, phosphorylation, ubiquitination, and methylation. Post‐translational acetylation and methylation of lysine residues on the N^*ε*^‐terminal tails of histones (Figure 1) are well‐characterized histone modifications that are read by bromodomains, chromodomains, tryptophan–aspartic acid repeats, and plant homeodomain (PHD) fingers.^[^ ^20^ ^]^ Histone acetylation is regulated by histone lysine acetyltransferases (KATs), which acetylate lysine, and HDACs, which remove acetyl groups from histone tails.^[^ ^1^ ^]^ Histone hyperacetylation is usually related to the “open” chromatin conformation, which is accessible to the transcriptional machinery. KATs neutralize the histone's positive charge by acetylating the N^*ε*^‐amino group of lysine residues and consequently weaken the interaction between DNA and histone.^[^ ^10^, ^21^ ^]^ KATs were mainly classified into the Gcn5 N‐acetyltransferases, MYST, and cAMP response element‐binding protein‐binding protein/E1A‐associated protein p300 (CBP/p300) families.^[^ ^21^ ^]^ In contrast, the removal of acetyl groups was catalyzed by HDAC, which resulted in chromatin condensation and gene silencing.^[^ ^10^, ^22^ ^]^ The HDAC eraser family is mainly divided into four types of enzymes, which include class I (HDAC 1, 2, 3, 8), class IIA (4, 5, 7, 9), class IIB (6, 10), class III (sirtuins 1–7), and class IV (HDAC11).^[^ ^23^ ^]^ Class I, II, and IV HDACs act on zinc (Zn)‐dependent catalytic mechanisms, whereas class III HDACs act on NAD^+^‐dependent catalytic mechanisms.^[^ ^21^, ^24^ ^]^ HDACs remove acetyl to compress the chromatin structure, which is associated with gene transcription inhibition. Consistent with this, HDACs have become a potential target for reversing epigenetic changes related to cancer, and several HDAC inhibitors (HDACi) have shown effective anticancer activities.^[^ ^25^ ^]^ Vorinostat (class I, II, and IV HDACi),^[^ ^26^ ^]^ belinostat (class I, II, and IV HDACi),^[^ ^27^ ^]^ and romidepsin (class I HDACi)^[^ ^28^ ^]^ have been granted FDA approval for peripheral and cutaneous T‐cell lymphoma treatment, and next‐generation chidamide (HDAC1, HDAC2, HDAC3, and HDAC10 inhibitors) has been approved by China FDA.^[^ ^29^ ^]^ Panobinostat (class I, II, and IV HDACi) combined with bortezomib and dexamethasone has also been approved for the treatment of multiple myeloma.^[^ ^30^ ^]^ Acetylation of lysine residues can be read by bromodomain and extraterminal (BET) proteins, which include bromodomain‐containing proteins (BRD2, BRD3, BRD4, and BRDt).^[^ ^21^ ^]^ In the clinic, some BET inhibitors have been developed and tested. Targeting BET is a promising strategy in cancer treatment. The BET inhibitors OTX015,^[^ ^31^ ^]^ and Molibresib (GSK525762)^[^ ^32^ ^]^ have recently been shown to generate considerable exciting activity in patients with cancer (Table 1). JQ1, a small‐molecule inhibitor of BET, can competitively inhibit the binding between acetylated histone and BRD4, which results in the inhibition of transcriptional regulatory function (Table 1).^[^ ^33^ ^]^

Histone methylation occurs in the basic amino acid side chains of lysine (Lys, K) and arginine (Arg, R) residues, which is performed by histone methyltransferase (HMT) utilizing SAM as the methyl donor.^[^ ^34^ ^]^ Histone lysine methyltransferases (KMTs) and protein arginine methyltransferases (PRMTs) are the major histone methyltransferases (HMTs). Histone lysines may be mono‐, di‐, or tri‐methylated.^[^ ^35^ ^]^ Histone methylation modification is involved not only in gene repression but also in gene activation.^[^ ^21^ ^]^ Almost all KMTs contain a conserved SET domain that was characterized as a suppressor of variegation 3–9, and enhancer of zeste and trithorax,^[^ ^36^ ^]^ except for disruptor of telomeric silencing 1‐like protein (DOT1L).^[^ ^14^, ^37^ ^]^ Several KMTs, including enhancer of zeste homolog 2 (EZH2) and DOT1L, have been developed as therapeutic targets.^[^ ^38^ ^]^ EZH2, the catalytic subunit of polycomb repressive complex 2 (PRC2), has been implicated in transcriptional repression by catalyzing the trimethylation of H3K27. The overexpression of EZH2 is related to tumorigenesis and the poor prognosis of several solid tumors.^[^ ^39^ ^]^ Tazemetostat is generally approved by the FDA as an EZH2 inhibitor,^[^ ^40^ ^]^ and other drugs (e.g., CPI‐1205) are in trials (NCT02395601) (Table 1). KDMs can be roughly divided into lysine‐specific demethylase (LSD or KDM) and Jumonji‐C (JmjC) domain‐containing family (JMJD).^[^ ^41^ ^]^ LSD1 (KDM1A), a flavin adenine dinucleotide‐dependent enzyme with high specificity for H3K4, can demethylate mono‐ or di‐methylated lysine.^[^ ^42^ ^]^ The overexpression of LSD1 indicates tumor progression and is a potential prognostic predictor.^[^ ^43^ ^]^ JMJD demethylases, with the conserved domain of the Fe^2+^ dioxygenase JmjC domain, were used for trimethyl lysine demethylation through the oxidative decarboxylation of *α*‐KG.^[^ ^44^ ^]^ ORY‐1001, a highly selective LSD1 inhibitor (LSD1i), has been reported to undergo clinical trials for leukemia and solid tumor patients.^[^ ^45^ ^]^

PRMTs can catalyze histone arginine methylation and have been implicated in various cellular processes, including signal transcription, DNA repair, gene transcription, and mRNA splicing.^[^ ^46^ ^]^ Arginine residues can be monomethylated, symmetrically, or asymmetrically dimethylated.^[^ ^47^ ^]^ Nine PRMTs catalyze the formation of three types of arginine methylation, including monomethylarginine (MMA), symmetric dimethylarginine (SMDA), and asymmetric dimethylarginine (ADMA),^[^ ^48^ ^]^ results in methylation of the guanidinium group.^[^ ^49^ ^]^ There are Type I (PRMT1, PRMT2, PRMT3, PRMT4, PRMT6, and PRMT8), II (PRMT5 and PRMT9), and III PRMT (PRMT7).^[^ ^46^ ^]^ The production of MMA is catalyzed by Type I, II, and III PRMT. Type I PRMT generate ADMA, while Type II PRMT catalyzes the production of SDMA.^[^ ^46^ ^]^ PRMTs play an important role in epigenetic regulation; however, overexpression of PRMTs is linked to poor prognosis of various cancers.^[^ ^46^ ^]^ Particularly, PRMT5 is overexpressed in a variety of tumors and has been considered the target for the anti‐tumor therapy strategy.^[^ ^50^ ^]^ In clinical trials, GSK3326595, a PRMT5 inhibitor, has been evaluated in breast cancer (NCT04676516) (Table 1).

#### 2.1.3 Non‐Coding RNAs

Although RNA is considered the main messenger of DNA translation into protein, non‐coding RNA (ncRNA), not involving in protein production, accounts for more than 90% of human genome‐derived RNA, and many ncRNAs have been proven to be closely related to the progression of cancer.^[^ ^51^ ^]^ ncRNAs have been identified as functional regulatory molecules that mediate chromatin remodeling, transcription, post‐transcriptional modification, and signal transduction.^[^ ^52^ ^]^ The ncRNAs can be categorized into small ncRNAs and long non‐coding RNAs (lncRNAs) according to their size. Transfer RNA‐derived small RNAs, microRNAs, and PIWI‐interacting RNAs are called small ncRNAs, whose size spectrum is less than 200 nt. In contrast, the ncRNAs with a length greater than 200 nt include lncRNAs, pseudogenes, and circular RNAs.^[^ ^51^ ^]^ Compared with small ncRNAs, lncRNAs appear to have more diverse mechanisms in transcriptional regulation. Multiple lncRNAs may act as flexible scaffolds for the chemical interactions of various chromatin regulators.^[^ ^21^ ^]^ The lncRNA homeobox transcript antisense RNA (HOTAIR) is one of the most well‐characterized lncRNAs that functions by directly combining with a protein complex. The expression of HOTAIR is abnormally elevated in breast cancer, lung cancer, and colorectal cancer.^[^ ^53^ ^]^ The 5' and 3' domains of HOTAIR are bound to the LSD1/CoREST/REST complex and the PRC2 complex, respectively, thereby targeting the complex to chromatin and coordinating increased H3K27me3 with decreased H3K4me3.^[^ ^54^ ^]^ Given their critical roles in cancer progression, ncRNAs are novel new targets for tumor microenvironment intervention.

### 2.2 Epigenetic Control of Gene Expression for Tumor Microenvironment Remodeling

#### 2.2.1 Epigenetic Regulation of Tumor‐Associated Macrophages

TAMs are abundant in the TME and participate in the regulation of cancer progression. TAMs are generally categorized into antitumor M1‐phenotype (TAM1) and protumor M2‐phenotype (TAM2).^[^ ^55^ ^]^ TAM repolarization can reverse the immunosuppressive microenvironment; and thus, enhance the anti‐tumor immune response.^[^ ^56^ ^]^ TAMs play a vital role in tumor growth,^[^ ^57^ ^]^ metastasis,^[^ ^58^ ^]^ and drug resistance.^[^ ^59^ ^]^ It is well established that macrophages are heterogeneous and plastic cells that respond to various TMEs. The phenotype of TAMs is regulated by epigenetic reprogramming. In addition, the recruitment and infiltration of TAMs are systemically affected by tumor cell‐derived pro‐inflammatory cytokines and chemokines through an epigenetic mechanism.

Epigenetic reprogramming is directly associated with the regulation of TAM phenotypes. Tumor‐derived exosomal miR‐138‐5p was involved in intercellular crosstalk between tumor cells and TAMs, and prompted the reprogramming of macrophages (from TAM1 to TAM2) through epigenetic regulation. miR‐138‐5p integrated into TAMs downregulated lysine demethylase 6B (KDM6B or JMJD3) expression, which elicited the enrichment of H3K27me3 in the promoter region encoding pro‐inflammatory genes; consequently resulting in the inhibition of TAM1, the activation of TAM2, and promoting the lung metastasis of breast cancer.^[^ ^60^ ^]^

The infiltration of TAMs is partly regulated by the epigenetic modification of tumor cells. The infiltration of TAMs was increased in breast cancer tissue, which was attributed to the fact that EZH2‐mediated epigenetic silencing of miR‐29b or miR‐30d promoted the expression of lysyl oxidase 4, which especially drove the tumorigenesis and metastasis of breast cancer cells through TAM2 activation and collagen remodeling.^[^ ^61^ ^]^ The histone demethylase JMJD1A (JHDM2A, KDM3A) drove tumor aggressiveness by promoting angiogenesis and TAM infiltration into tumor tissue under hypoxia and nutrient starvation. JMJD1A inhibition overcame resistance to antiangiogenic treatments and enhanced antitumor effects.^[^ ^62^ ^]^ In addition, epigenetic modification of tumor cells could control the recruitment and infiltration of TAMs by regulating pro‐inflammatory cytokine and chemokine pathways. Macrophage infiltration in small‐cell lung cancer was significantly reduced because the epigenetic‐dependent silencing of chemokine (C‐C motif) ligand 2 (CCL2) in tumor cells impeded the recruitment of macrophages derived from blood monocytes into tumor tissue. EZH2‐mediated H3K27me3 and DNMT1‐mediated DNA hypermethylation were attributed to the epigenetic silencing of CCL2, which could be reversed by the combined treatment of EPZ011989 (EZH2 inhibitor) and decitabine (DNMT1 inhibitor); thus, facilitating TAM1 infiltration and tumor killing.^[^ ^63^ ^]^ Moreover, a recent study demonstrated that lysine‐specific demethylase 6A (KDM6A) repolarized TAMs by regulating the expression of CCL2 and IL‐6. KDM6A functions as a demethylase for H3K27 and is a component of a complex of proteins associated with Set 1‐like complexes for promoting H3K4me1 and H3K27ac. KDM6A deficiency endowed cancer stem cell characteristics and promoted the growth of bladder cancer cells by enhancing M2 polarization through the activation of pro‐inflammatory cytokine (IL‐6) and chemokine pathways (CCL2) indirectly due to the increase in H3K27me3 and the decrease in H3K4me1.^[^ ^57^ ^]^ Furthermore, TAMs, recruited by lncRNAs‐driven CCL2, play a vital role in tumor metastasis. Lymph node metastasis‐associated transcript 1 (LNMAT1), termed a class of lncRNAs, was proposed to promote lymph node metastasis in bladder cancer. The mechanism described is that LNMAT1 epigenetically activated tumor cell‐derived CCL2 and enhanced transcription by promoting hnRNPL‐mediated H3K4me3 at the promoter of CCL2, which distinctly recruited macrophages into tumors. TAM infiltration into tumor tissue contributed to lymphangiogenesis and lymphatic metastasis by producing vascular endothelial growth factor C (VEGF‐C), a class of lymphangiogenesis growth factors.^[^ ^58^ ^]^ In addition, the drug resistance mechanism of tumors is associated with CCL2‐mediated macrophage remodeling involving epigenetic alteration. KRAS mutation‐independent pancreatic ductal adenocarcinoma (PDAC) recurrence has been proposed to involve an HDAC5‐driven epigenetic mechanism. HDAC5 inhibited the expression of suppressor of cytokine signaling 3 (SOCS3) through histone deacetylation of H3K9ac and H3K27ac, and subsequently, the repression of SOCS3 further negatively regulated the expression of CCL2 and positively enabled the conversion from neutrophils to CCR2^+^ macrophages, which in turn provided support for the proliferation of cancer cells through KRAS mutations independent of transforming growth factor‐beta 1 (TGF‐*β*)/SMAD4. The synergistic inhibition of KRAS and TGF*β*/SMAD4 signaling pathways may damage the growth of KRAS mutation‐dependent tumors and alleviate TGF*β*‐induced immunosuppression.^[^ ^59^ ^]^

TAMs regulate tumor metabolism through an epigenetic mechanism. Metabolic reprogramming of cancer cells is an important hallmark of solid tumor progression. Aerobic glycolysis conferred apoptosis resistance in breast cancer cells. TAMs elevated aerobic glycolysis and chemoresistance of tumor cells through hypoxia‐inducible factor‐1 (HIF‐1)‐stabilizing long noncoding RNA (HISLA) transmitted by extracellular vesicles. HISLA restrained the interaction between PHD2 and HIF‐1*α* to accelerate the accumulation of HIF‐1*α* and the release of lactic acid from tumor cells, which in turn up‐regulated HISLA in macrophages as a positive feedback loop.^[^ ^64^ ^]^

Overall, these studies highlight the crosstalk between tumors and TAMs involving epigenetic regulation. So, we propose that epigenetic therapy might be used to intervene in the recruitment, infiltration, and repolarization of macrophages to overcome tumor growth, metastasis, and drug resistance.

#### 2.2.2 Epigenetic Regulation of T Lymphocytes

T cells play a major role in adaptive immunity by secreting soluble mediators or cell contact. T cells are characterized by enormous plasticity and can differentiate into different T‐cell subsets in response to microenvironments. Naive T cells emerged from the thymus monitored major histocompatibility complex (MHC) molecules.^[^ ^65^ ^]^ Once activated by antigen‐presenting cells (APCs), naive T cells can rapidly differentiate into effector T cells,^[^ ^66^ ^]^ regulatory T cells (Treg),^[^ ^67^ ^]^ cytotoxic T cells (CTL),^[^ ^68^ ^]^ or memory T cells.^[^ ^66^ ^]^ CD4^+^ T helper cells, including Th1 cells, Th2 cells, Treg cells, and Th17 cells,^[^ ^69^ ^]^ provoke an immune response by activating other immune cells, while CD8^+^ CTLs mainly secrete perforin and granzyme for direct killing.^[^ ^70^ ^]^ A majority of tumor patients lack a lasting response to immunotherapy, which is partly caused by epigenetic‐mediated T‐cell dysfunction.^[^ ^71^ ^]^ Understanding the epigenetic regulation of the phenotype and function of T cells as well as the tumor cell‐mediated Th1 chemokines is crucial for impeding immune evasion when considering epigenetic therapy for cancer treatment.

Epigenetic reprogramming can directly change the phenotype and function of T cells. De novo DNA methylation aggravated T‐cell exhaustion, whereas methylation inhibition displayed T‐cell regeneration. Recent studies have indicated that the DNA‐demethylating agent decitabine‐treated chimeric antigen receptor T cells restricted the expression of exhaustion‐associated genes, and enhanced the expression of memory‐related markers, which were associated with enhanced antitumor functionality.^[^ ^72^ ^]^ Moreover, low‐dose decitabine therapy inhibited cancer progression by facilitating the production of IFN‐*γ* ^+^ Th1 cells and CTLs.^[^ ^73^ ^]^ Epigenetic regulation of the transcription factor Bhlhe40 programs tissue‐resident memory CD8^+^ T cells and CD8^+^ tumor‐infiltrating lymphocytes; thus, promoting tumor control. Limited nutrients in the tumor cause stress to resident CD8^+^ T cells. Bhlhe40, a stress‐responsive transcription factor, provoked the genetic characteristics of tissue‐resident memory CD8^+^ T cells and CD8^+^ tumor‐infiltrating lymphocytes associated with mitochondrial fitness and functional epigenetic states through epigenetic and metabolic mechanisms. Bhlhe40 deficiency reduced the metabolite production required for acetyl‐CoA synthesis and histone H3 acetylation in CD8^+^ T cells, resulting in impaired effector molecule expression and antitumor efficiency. Epigenetic modulators combined with certain metabolites potentiated CD8^+^ T‐cell‐dependent tumor control.^[^ ^74^ ^]^ Furthermore, NR4A1 induced T cell dysfunction through an epigenetic mechanism. Tolerant T cells, characterized by maintaining T cells unresponsive to self‐tissue, highly expressed the transcription factor NR4A1, which inhibited effector gene expression through the considerable inhibition of AP‐1 function and positively activated tolerance‐related genes through the hyperacetylation of H3K27ac, thereby restraining the differentiation of effector T cells. Therefore, NR4A1 is considered a target for cancer immunotherapy.^[^ ^75^ ^]^ In addition, HDAC3 inhibited gene expression involved in the CD8 T cell effector and cytotoxicity differentiation. HDAC3 negatively regulated genes (e.g., Runx3 and Prdm1) in CD8 T cells through the deacetylation of H3K27ac of transcription factors involved in CD8 T cell activation and differentiation; thus, inhibiting the differentiation of T cells into cytotoxic effector T cells.^[^ ^76^ ^]^ Taken together, these studies further highlighted the important role of DNA methylation and histone modification in T‐cell differentiation.

Epigenetic changes in cancer cells may cause immune evasion. A novel immune escape mechanism in which epigenetic silencing of Th1‐type chemokines produced by tumors could repress T‐cell tumor homing was proposed. Modification of DNA methylation plays a critical role in the regulation of gene expression. Chemokines are key factors of T‐cell homing in tumors; the transcriptional repression driven by DNA methylation can affect the expression of chemokines. A recent study highlighted that chemokines mediate the accumulation of T cells in solid tumors and improve the immunoreactivity of tumors. CCL5 derived from tumor cells and C—X—C motif chemokine ligand 9 (CXCL9) secreted by IFN‐*γ*‐stimulated macrophages and DCs were critical for CD8^+^ T‐cell recruitment. Overexpression of CCL5 and CXCL9 could convert “cold” tumors into “hot” tumor immunophenotypes, whereas epigenetic silencing caused by DNA hypermethylation in human tumors induced a decrease in CCL5 expression, leading to tumor immune evasion.^[^ ^77^ ^]^ Nevertheless, DNA hypomethylating agent treatment promoted the CD8^+^ T‐cell infiltration into tumor tissue to boost the antitumor immune response.^[^ ^78^ ^]^ In one study, epigenetic reprogramming of tumor cells may drive Th1 chemokine‐mediated effector T‐cell trafficking. PRC2 restrained gene transcription by trimethylating H3K27. PRC2 expression was negatively associated with effector T‐cell trafficking in colon cancer tissue. The PRC2 complex and JMJD3‐mediated H3K27me3 inhibited the expression of CXCL9 and CXCL10, which controlled CD4^+^ and CD8^+^ T‐cell tumor trafficking.^[^ ^79^ ^]^ In another study, osteosarcoma epigenetically downregulated CXCL12 expression through DNMT1‐induced hypermethylation, which consequently endowed osteosarcoma with the characteristics of metastasis and restrained cytotoxic T‐cell tumor trafficking driven by Th1‐type chemokines secretion in an indirect CXCL12‐dependent manner (e.g., CXCL9 and CXCL10), while decitabine treatment significantly enhanced CXCL12 expression in tumors, resulting in boosting of the immune response and prevention of metastasis.^[^ ^80^ ^]^ Furthermore, EZH2‐mediated H3K27me3 and DNMT1‐mediated DNA hypermethylation independently regulated Th1‐type chemokine (CXCL9 and CXCL10) repression in the tumor and consequently restrained intratumoral CD8^+^ T cells. Treatment with EZH2 and DNMT1 epigenetic regulators synergistically loosened Th1‐type chemokine repression and promoted effector T‐cell tumor trafficking; thus, ultimately potentiating the therapeutic effect of programmed death‐ligand 1 (PD‐L1) and adoptive T‐cell transfusion.^[^ ^81^ ^]^

This process of naive T cells activated by antigen presentation in tumors can be blocked in an epigenetic modification‐dependent manner. For example, the loss of MHC class I (MHC‐I) antigen presentation in cancer cells enabled immune evasion through epigenetic regulation. The transcriptional silencing of the MHC‐I antigen processing pathway (MHC‐I‐APP) was mediated by an epigenetic repressive complex PRC2 that silenced the critical genes necessary for MHC‐I‐APP through the enrichment of bivalent activation H3K4me3 and inhibitory H3K27me3 in the MHC‐I‐APP gene promoters. Importantly, PRC2 inhibitors alleviated the transcriptional inhibition of MHC‐I to rebuild CD8^+^ T cell‐mediated antitumor immunity.^[^ ^82^ ^]^ Moreover, BRD4 inhibitors (BRD4i, e.g., JQ1) decreased PD‐L1 expression and increased MHC‐I expression on prostate cancer cells, which led to enhanced tumor immunogenicity and intratumoral CTL infiltration; thus, finally resensitizing pancreatic cancer to immune checkpoint blockade (ICB) therapy.^[^ ^83^ ^]^

Overall, a better understanding of T‐cell‐specific epigenetic mechanisms and the crosstalk between epigenetic dysregulation of tumor cells and T cells may help reshape antitumor immunity.

#### 2.2.3 Epigenetic Regulation of Cancer‐Associated Fibroblasts

As the main types of stromal cells in the TME, CAFs have been proven to be correlated with the proliferation, invasion, migration, and drug resistance of tumors.^[^ ^84^ ^]^ The epigenetic reprogramming of CAFs and the crosstalk between cancer cells and CAFs can be driven by various mechanisms.

The epigenetic regulation of fibroblasts is critical for cancer progression. The proinflammatory leukemia inhibitory factor reprogramed fibroblasts into aggressive CAFs through the activation of Janus kinase 1/ signal transducer and activator of transcription 3 (JAK1/STAT3), which was controlled by epigenetic changes. In detail, the activation of JAK1/STAT3 was initiated by enhanced p300 (histone acetyltransferase)‐mediated STAT3 acetylation, which, in turn, promoted the phosphorylation of JAK1 through DNMT3b hypermethylation‐dependent *Src* homology region 2 domain‐containing phosphatase‐1 (SHP‐1) silencing, whereas the sustained activation of JAK1/STAT3 signaling was maintained by DNMT1.^[^ ^85^ ^]^ Beside, the relationship between abnormal metabolism and epigenomic alterations of CAFs has emerged as a threat to cancer progression. In one study, the PDAC cell‐secreted lactic acid drove epigenetic alteration of CAFs; thus, promoting the invasion of PDAC. Pancreatic CAFs are derived from the differentiation of pancreatic stellate and mesenchymal stem cells.^[^ ^86^ ^]^ The paracrine lactic acid secreted by PDAC cells could lead to widespread demethylation during the transformation of mesenchymal stem cells to CAFs by increasing *α*‐KG, which activated the demethylase TET. The increased hydroxymethylcytosine in CAFs promoted epigenetic reprogramming, which was associated with the upregulation of the transcript encoding CXCR4, which in turn promoted the invasion of PDAC.^[^ ^87^ ^]^ In another study, the epigenetic alteration of CAFs could possibly potentiate glutamine metabolism and contribute to prostate cancer progression. The neuroendocrine differentiation of epithelial cells mediated by epigenetic modification of CAFs was sufficient to promote the resistance of prostate cancer to androgen deprivation therapy. Hypermethylation and gene silencing of the RAS protein activator‐like 3 promoter in prostate cancer‐related CAFs induced RAS‐dependent macropinocytosis for the uptake of albumin, which generated glutamine via lysosomal degradation. Glutamine was localized to epithelial cells for TCA recycling and mTOR activation; thus, leading to cancer progression. The combination of androgen receptor antagonists and targeted therapy of glutamine uptake by cancer cells can be used as a synthetic method to prevent prostate cancer growth.^[^ ^84b^ ^]^ Furthermore, the glucose metabolism of CAFs changes during tumor progression. Glycolysis of CAFs mediated by oxygen‐dependent epigenetic modification fueled breast cancer cells and facilitated tumor growth. CAF showed enhanced glycolysis activity, which was maintained by epigenetic reprogramming of HIF‐1*α*, PKM, and LDHA with hypomethylation.^[^ ^88^ ^]^ Consistent with these effects, the regulation of proinvasive CAFs by epigenetic drugs is considered an anticancer treatment strategy. The selective poor response of squamous cell carcinoma (SCC)‐CAF to the anti‐fibrotic drug nintedanib is available through the TGF*β* transcription factor SMAD3 repression, which is driven by SMAD3 promoter hypermethylation. Epigenetic inhibition of SMAD3 in SCC‐CAFs treated with the globalized demethylating agent 5‐AZA may rescue nintedanib clinical failure in SCC.^[^ ^89^ ^]^ Scriptaid, a selective inhibitor of HDACs 1/3/8, could control the growth and invasion of tumors by reversing tumor‐supportive features of CAF through epigenetic regulation.^[^ ^84c^ ^]^

CAFs can drive epigenetic regulation of cancer cells through various mechanisms; thus, prompting the progression and metastasis of tumors. CAFs interact with tumor cells through paracrine mechanisms, which can favor breast cancer metastasis. TGF‐*β*1 secreted by CAFs was responsible for activating the transcription of HOTAIR in tumor cells, which resulted in significantly increased H3K27me3 levels on the CDK5RAP1 and EGR‐1 promoters, which in turn activated CDK5‐mediated epithelial–mesenchymal transition and breast cancer metastasis.^[^ ^84a^ ^]^ The insight into the process of CAF‐driven epigenetic proliferation of carcinoma cells has revealed that ECM‐CAF‐driven JMJD1A (KDM3A, an H3K9‐specific demethylase) in tumor cells positively regulates Yes‐associated protein/transcriptional coactivator with PDZ‐binding motif (YAP/TAZ, mechanosensitive regulators of cell proliferation) transcription in a stiffness‐dependent manner.^[^ ^90^ ^]^

Overall, with a greater understanding of the interaction between direct and indirect effects of epigenetic regulation on CAF‐mediated protumor features, there is an alternative strategy for further developing epigenetic agents targeting CAF to regulate the progression and metastasis of tumors.

## 3 Epigenetic Modulator‐Based Nanotechnology for Improving Tumor Therapy

**Figure 2:**
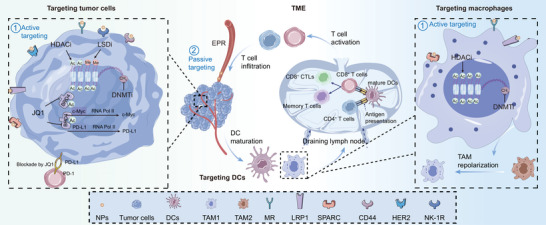
Epigenetic modulator‐based nanotechnology for regulating TME by targeting tumor cells and APCs (e.g., macrophages and DCs). Epigenetic agent‐based nanomedicine may target tumor cells or APCs (including macrophages and DCs) after systemic delivery with the aid of EPR effect‐mediated passive targeting or ligand‐mediated active targeting. Epigenetic drugs including DNMTi, HDACi, BRD4i, and LSD1i, directly target tumor cells for regulating apoptosis, proliferation, migration, and therapy resistance, as well as for tumor microenvironment (TME) remodeling by inducing DC maturation and T cell activation and infiltration. Moreover, epigenetic modulator‐based nanotechnology can facilitate tumor‐associated macrophage (TAM) polarization by targeting macrophages and induce DC maturation by targeting DCs.

**Figure 3:**
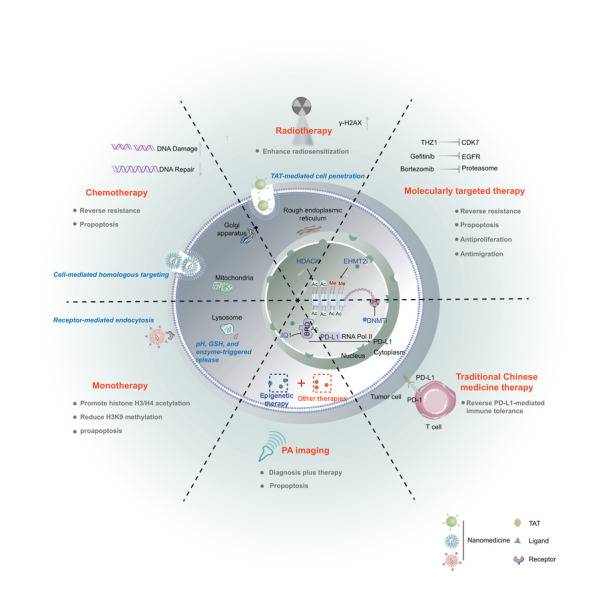
Epigenetic agent‐containing combination therapies target tumor cells for regulating apoptosis, proliferation, migration, and therapy resistance. Specific delivery of nanomedicine can be achieved via various strategies, including cell‐mediated homologous targeting, receptor‐mediated endocytosis (e.g., CD44 and *α*v*β*3 integrin receptors), and TAT‐mediated cell penetration (e.g, cell‐penetrating peptide derived from the transactivator of transcription of HIV‐1). After delivery to tumor tissue, epigenetic drugs and other antitumor drugs can be released from nanoplatforms in response to the TME (e.g., pH, GSH, and enzyme) to exert optimal synergetic effects. The combination of epigenetic therapy and other antitumor therapies (including chemotherapy, radiotherapy, molecularly targeted therapy, traditional Chinese medicine therapy, and PA imaging) shows remarkable efficiency in the treatment of solid tumors through multiple mechanisms. Ligand, HA and iRGD; PA, photoacoustic imaging; PD‐1, programmed cell death protein 1; PD‐L1, programmed cell death‐ligand 1; Receptor, CD44 and *α*v*β*3 integrin receptors; TAT, cell‐penetrating peptide.

**Figure 4:**
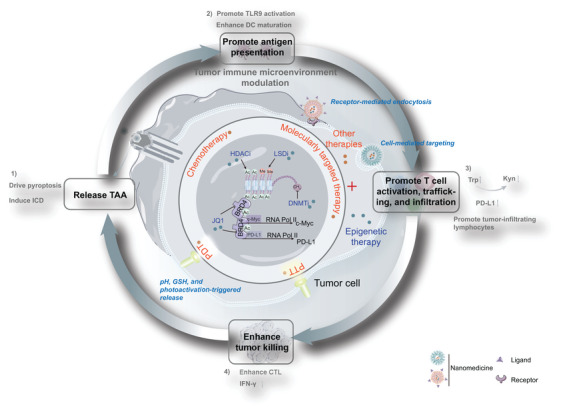
Epigenetic agent‐containing combination nanomedicines target tumor cells for tumor immune microenvironment modulation. Nanomedicine can actively accumulate in the tumor cells via cell‐mediated homologous targeting and nonhomologous targeting (e.g., T lymphocyte membrane) as well as receptor‐mediated endocytosis (e.g., CD44). Epigenetic drugs and other antitumor drugs released from nanomedicine respond to TME (e.g., pH, GSH, and photoactivation‐triggered release). Epigenetic monotherapies or synergy with other therapies (including chemotherapy, molecularly targeted therapy, PTT, and PDT) facilitate the cancer‐immunity cycle by promoting the release of TAA, antigen presentation, T cell activation, trafficking, and infiltration; thus, exerting tumor killing. More specifically, 1) the release of TAAs are initiated by pyroptosis and ICD, 2) the antigen presentation ability of DCs is facilitated through TLR‐9 activation and DC maturation, 3) the activation, trafficking, and infiltration of T cells are enhanced by inhibiting PD‐L1‐mediated immune tolerance and Trp metabolism as well as increasing tumor‐infiltrating lymphocytes, 4) tumor killing is achieved by CTL and IFN‐*γ*. CTL, cytotoxic T cell; ICD, immunogenic cell death; Kyn, kynurenine; Ligand, HA; Receptor, CD44; PDT, photodynamic therapy; PTT, photothermal therapy; TLR9, toll‐like receptor‐9; TAA, tumor‐associated antigen; and Trp, tryptophan.

**Figure 5:**
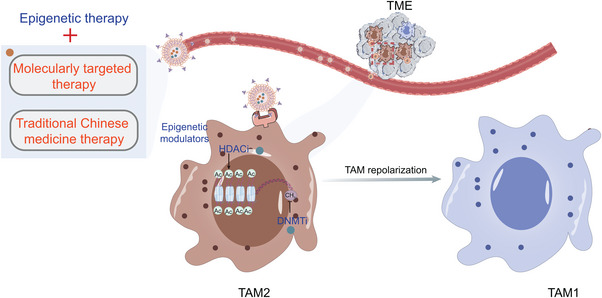
Epigenetic agent‐containing combination nanomedicines regulate macrophage repolarization. Epigenetic agent‐containing combination nanomedicines can be actively transported into macrophages in the TME through leaky tumor vasculature and receptors on the surface of macrophages. The combination of epigenetic therapy and other antitumor therapies (e.g., molecularly targeted therapy and traditional Chinese medicine therapy) synergistically enhances antitumor immunity and effectively inhibits the progression of tumors by repolarizing TAM2 to TAM1.

An increasing number of epigenetic modulators (e.g., DNMTi, HDACi, BRD4i, and LSD1i) are used in the development of accurate nanomedicines for the regulation of the TME.^[^ ^8^ ^]^ Epigenetic therapy potentiates the activity of other therapies. Preclinical and clinical evidence show that epigenetic modulators in combination with other therapies (including immunotherapy, chemotherapy, radiotherapy, hormone therapy, and molecularly targeted therapy) can improve the therapeutic effect on solid tumor patients.^[^ ^8^ ^]^ Herein, this review summarized the use of epigenetic modulator monotherapies or in combination with other anticancer therapies (including chemotherapy, radiotherapy, molecularly targeted therapy, photothermal therapy, photodynamic therapy, photoacoustic imaging, immunotherapy, hormone therapy, and other therapies) for improving the sensitivity of cancer cells. Epigenetic agents are plagued by poor water solubility, rapid clearance, and poor tissue distribution.^[^ ^91^ ^]^ Given that the greater permeability of tumor vessels than normal vessels and the impaired lymphatic flow, nanomedicines accumulate preferentially in tumor tissue and remain for a long time, which is called the enhanced permeability and retention (EPR) effect.^[^ ^92^ ^]^ Nanomedicine delivery strategies can utilize EPR effect‐mediated passive targeting or ligand‐mediated active targeting to deliver nanomedicines to tumor tissue after systemic delivery.^[^ ^93^ ^]^ Nanotechnology has been characterized as a particularly attractive strategy for accurate tumor delivery of one or several different anticancer drugs (**Table** **2**). With the aid of nanotechnology, epigenetic modulators may directly target tumor cells or target APCs (including macrophages and DCs) (**Figure** **2**, **Table** **3**). Epigenetic modulator monotherapies or in combination with other anticancer therapies can regulate apoptosis, proliferation, migration, and therapy resistance by targeting tumor cells (**Figure** **3**), as well as remodel tumor immune microenvironment by targeting tumor cells (**Figure** **4**) and APCs (**Figure** **5**).

**Table 2:** Epigenetic modulator‐based nanomedicines for improving tumor therapy

Delivery mechanism	Combination type	Nanoplatforms	Payload 1 (epigenetic modulator)	Payload 2	Combine	Ligand type	Target	Cancer type	Delivery method	Ref.
Active targeting	Epigenetic therapy	HRM/PLGA/DOTAP/SAHA NPs	SAHA	—	—	Hybrid membranes	Tumor	Metastatic lung cancer	iv	[94]
		HA‐VRS‐SLNs	SAHA	—	—	HA	Tumor‐CD44	Breast cancer/NSCLC/SCC	iv	[95]
		BSA‐ORY@TM/cM70 NPs	ORY‐1001	—	—	M70 and PD1‐CTLL‐2 membrane proteins	Tumor	TNBC/colon cancer/melanoma	iv	[114]
		SP‐PEG‐PDLLA‐ARV‐825 micelles	ARV‐825	—	—	Substance P peptide	Tumor‐NK‐1R	Glioma	iv	[124]
		LF‐Pan/JQ1 lipos	Panobinostat/JQ1	—	—	LF	Tumor/TAM‐LRP1/SPARC	Colorectal cancer	iv	[125]
	Epigenetic therapy/chemotherapy	CCMP‐DOX/SAHA Lipos	SAHA	Dox	—	Cancer cell membrane proteins	Tumor	NSCLC	iv	[102]
		DOX/siGCN5@HPMSNs	siGCN5	Dox	—	HA	Tumor‐CD44	Breast cancer	iv	[101]
	Epigenetic therapy/molecularly targeted therapy	HA‐PBLG‐Gef/SAHA NPs	SAHA	Gefitinib	—	HA	Tumor‐CD44	NSCLC	Intrapulmonary delivery/iv	[108]
		CS‐I/J@CM NPs	JQ1	Indoximod	Laser irradiation	Cancer cell membrane proteins	Tumor	Glioblastoma	Iv	[116]
		HCC‐PLGA‐BFA/JQ1 NPs	JQ1	Brefeldin A	—	Cancer cell membrane proteins	Tumor/ER‐Golgi apparatus	Melanoma	iv	[117]
		Tra/Man‐Gef/SAHA lipos	SAHA	Gefitinib	—	Trastuzumab/Mannose	Tumor‐HER2/TAM‐MR	NSCLC	iv	[126]
	Epigenetic therapy/photodynamic therapy	HA‐CD‐PPa/JQ1 NPs	JQ1	Pyropheophorbide a	Laser irradiation	HA	Tumor‐CD44	PDAC	iv	[122]
		BNPs	Decitabine	ICG	Laser irradiation	4T1 cancer cell membrane (integrin *α*4*β*1)	Tumor	Breast cancer	iv	[123]
	Epigenetic therapy/photoacoustic imaging	M1‐EM‐SUCS NPs	SAHA	*β*‐NaYF4:Er3+,Yb3+ UCs	Laser irradiation	M1‐derived exosome	Tumor‐VCAM1	Lung cancer	iv	[112]
	Epigenetic therapy/traditional therapy	iRGD‐PSS@PBAE@JQ1/ORI NPs	JQ1	Oridonin	—	iRGD	Tumor‐*α*v*β*3 integrin receptors	Breast cancer	iv	[111]
		Man‐LF‐SHK/JQ1 NPs	JQ1	Shikonin	—	MR/LF	Tumor/TAM‐MR/LRP1	Colon cancer	iv	[55]
Passive targeting	Epigenetic therapy	AZA‐PLGA‐PEG nano‐micelles	5‐AZA	—	—	—	Tumor	Breast cancer	ip	[96]
		ND‐UNC0646 complexes	UNC0646	—	—	—	Tumor	HCC	iv	[97]
		SAHA‐Zn^2+^‐5‐Aza nanofibers	SAHA/5‐AZA	—	—	—	Tumor	Gastric cancer	iv	[98]
	Epigenetic therapy/ chemotherapy	CDDP‐SAHA conjugated nanomicelles	SAHA	Cisplatin	—	—	Tumor	NSCLC	iv	[103]
		5‐FU/DAC‐PEG‐pep‐PCL NPs	Decitabine	5‐fluorouridine	—	—	Tumor	Gastric cancer	—	[104]
		LAQ/DOX‐DBHD NPs	LAQ824	DOX	—	—	Tumor	Breast cancer/SCC	iv	[115]
	Epigenetic therapy/radiotherapy	PLGA‐lecithin‐PEG NPs	SAHA or quisinostat	—	Laser irradiation	—	Tumor	Prostate and colorectal cancers	iv	[105]
		CAT‐SAHA@PLGA NPs	SAHA	Catalase	Laser irradiation	—	Tumor	Colorectal cancer	iv	[106]
		TAT‐Pt/SAHA‐PLGA NPs	SAHA	Pt^IV^	Laser irradiation	—	Tumor	Breast cancer	iv	[107]
	Epigenetic therapy/molecularly targeted therapy	JQ1/THZ1@8P4 NPs	JQ1	THZ1	—	—	Tumor	PDAC	iv	[109]
		ZNP/VB NPs	SAHA	Bortezomib	—	—	Tumor	Prostate cancer	iv	[110]
		PHPNJ NPs	JQ1	NLG919/ PPa	Laser irradiation	—	Tumor	Breast/colorectal cancer	iv	[118]
		AOZN nanomicelles	*γ*‐oryzanol	AMPCP	—	—	Tumor	Melanoma	iv	[119]
	Epigenetic therapy/photothermal therapy	CuS/CpG/JQ1 lipos	JQ1	CuS/CpG	Laser irradiation	—	Tumor	Breast cancer/PDAC	iv	[120]
	Epigenetic therapy/immunotherapy	CHI/BMS‐202@lipos	Chidamide	BMS‐202	—	—	Tumor	Breast cancer	iv	[127]
	Epigenetic therapy/ hormone therapy	POEG‐co‐PVDSAHA/TAM nanomicelles	SAHA	Tamoxifen	—	—	Tumor	TNBC	iv	[113]
Local delivery	Epigenetic therapy	PEGylated CDN NPs	Panobiostat	—	–	—	Tumor	Diffuse intrinsic pontine glioma	Intratumoral convection‐enhanced delivery	[91a]
		Bel‐PGON NPs	Belinostat	—	—	—	Tumor	Bladder cancer	Intratumoral delivery	[99]
	Epigenetic therapy/photothermal therapy	PDMN‐JQ1 NPs	JQ1	—	Laser irradiation	—	Tumor	TNBCs	Intratumoral injection	[121]

**Table 3:** Epigenetic nanomedicines targeting both the tumor cell and TME

Nanoplatforms	Epigenetic modulator	Epigenetic modulator targets	Epigenetic modulator mechanisms	Ref.
HRM/PLGA/DOTAP/SAHA NPs	SAHA	NSCLC cells (NCI‐H1299 cells)	Promoted histone acetylation driven‐apoptosis	[94]
HA‐VRS‐SLNs	SAHA	NSCLC cells (A549 cells), SCC cells (SCC‐7 cells), and breast cancer cells (MCF7 cells)	—	[95]
AZA‐PLGA‐PEG nano‐micelles	5‐AZA	Breast cancer cell (MCF7 cells)	DNA incorporation	[96]
ND‐UNC0646 complexes	UNC0646	HCC cell (SNU‐398 cells)	Reduced H3K9 methylation	[97]
SAHA‐Zn^2+^‐5‐Aza nanofibers	SAHA/5‐AZA	Gastric cancer cells (MGC‐803 cells)	Increased epigenetic reprogramming‐mediated proapoptosis	[98]
PEGylated CDN NPs	Panobiostat	Glioma cells (GL26 cells)	—	[91a]
Bel‐PGON NPs	Belinostat	Bladder cancer cells (T‐24 cells and UM‐UC‐3 cells)	Increased acetylated‐histone H4	[99]
CCMP‐DOX/SAHA Lipos	SAHA	NSCLC cells (NCI‐H1299 cells)	Increased acetylated histone H3	[102]
DOX/siGCN5@HPMSNs	siGCN5	Drug‐resistant breast cancer cells (MCF7/ADR cells)	Abolited P‐gp‐mediated DOX resistance	[101]
CDDP‐SAHA conjugated nanomicelles	SAHA	NSCLC cells (A549/DR cells)	Reversed cisplatin resistance	[103]
5‐FU/DAC‐PEG‐pep‐PCL NPs	Decitabine	Gastric cancer cells (MKN45 cells)	Restored TFAP2E to re‐sensitize cancer cells to chemotherapy	[104]
PLGA‐lecithin‐PEG NPs	SAHA or quisinostat	Prostate cancer cells (PC3 cells) and colon cancer cells (SW620 cells)	Enhanced *γ*‐H2AX‐mediated radiosensitization	[105]
CAT‐SAHA@PLGA NPs	SAHA	Colon cancer cells (CT26 cells)	Enhanced *γ*‐H2AX‐mediated radiosensitization	[106]
TAT‐Pt/SAHA‐PLGA NPs	SAHA	Breast cancer cells (EMT‐6 cells)	Enhanced *γ*‐H2AX‐mediated radiosensitization	[107]
HA‐PBLG‐Gef/SAHA NPs	SAHA	NSCLC cells (A549 cells)	Pro‐apoptosis	[108]
JQ1/THZ1@8P4 NPs	JQ1	PDAC cells (BxPC‐3, PANC‐1, and PDX0032 cells)	Suppressed SE‐associated oncogenic transcription to alleviate gemcitabine‐resistant PDAC	[109]
ZNP/VB NPs	SAHA	Prostate cancer cells (PC3, DU145, and LNCap cells)	Driven ER stress‐mediated apoptosis	[110]
iRGD‐PSS@PBAE@JQ1/ORI NPs	JQ1	Breast cancer cells (4T1 cells)	Reversed PD‐L1‐mediated immune tolerance	[111]
M1‐EM‐SUCS NPs	SAHA	Lung cancer cells (LLC and A549 cells)	Promoted histone acetylation driven‐apoptosis	[112]
POEG‐co‐PVDSAHA/TAM nanomicelles	SAHA	TNBC cells (MDA‐MB‐231, HS578T, and 4T1.2 cells)	Activated ER*α* to re‐sensitize TNBCs to TAM therapy	[113]
BSA‐ORY@TM/cM70 NPs	ORY‐1001	TNBC cells (4T1 cells)	Induced IFN‐*γ*‐mediated tumor‐infiltrating lymphocyte accumulations. BSA‐ORY@TM/cM70 NPs increased mature DCs, intratumoral CD8^+^ T cells, Ki‐67^+^ CD8^+^ T cells, and GzmB^+^ CD8^+^ T cells in orthotopic 4T1 bearing mice. (TME)	[114]
LAQ/DOX‐DBHD NPs	LAQ824	Breast cancer cells (4T1 cells) and head and neck SCC cells (SCC7 cells)	Induced pyroptosis to alleviate the TIME. LAQ824 induced DC maturation, fewer Tregs, and MDSCs in vivo. (TME)	[115]
CS‐I/J@CM NPs	JQ1	Glioblastoma cells (GL261 cells)	Inhibited PD‐L1‐mediated immunosuppression. CS‐I/J@CM NPs + laser reprogramed TAMs and increased DC maturation in vitro, induced memory T cells, and CD8^+^ T cell infiltration, DC maturation, and Treg inhibition in vivo. (TME)	[116]
HCC‐PLGA‐BFA/JQ1 NPs	JQ1	Melanoma cells (B16‐F10 cells)	Inhibited PD‐L1‐mediated immunosuppression. HCC@NP‐JQ1 increased CD8/Treg ratio in vivo. (TME)	[117]
PHPNJ NPs	JQ1	Breast cancer cells (4T1 cells) and colorectal cancer cells (CT26 cells)	Inhibited PD‐L1‐mediated CTL exhaustion. PHPNJ NPs + laser remarkably elicited DC maturation, tumor‐infiltrating CD3^+^ T cells, CD8^+^ T cells, and effector T cells, and depleted Tregs in 4T1 and CT26 tumor‐bearing mice. (TME)	[118]
AOZN nanomicelles	*γ*‐oryzanol	Melanoma cells (B16‐F10 cells)	Induced pyroptosis to alleviate the TIME. *γ*‐oryzanol promoted CD8 T cell infiltration and DC maturation, and inhibited MDSC accumulation and TAM2 polarization in vivo. (TME)	[119]
CuS/CpG/JQ1 lipos	JQ1	PDACs (Panc02 cells)	Inhibited PD‐L1‐mediated immunosuppression. CuS/CpG/JQ1 lipos increased CD8^+^ T cells/CD3^+^ T cells ratio in Panc02 tumor‐bearing C57BL/6 mice. (TME)	[120]
PDMN‐JQ1 NPs	JQ1	TNBCs cells (4T1 cells)	Inhibited c‐Myc/PD‐L1‐mediated immunosuppression. JQ1 increased CD8, CD3, and central memory T cells in the primary and distant tumor in vivo. (TME)	[121]
HA‐CD‐PPa/JQ1 NPs	JQ1	PDACs (Panc02 cells)	Inhibited c‐Myc‐initiated glycolysis and PD‐L1‐mediated immunosuppression. HA‐CD‐PPa/JQ1 NPs + laser promoted DCs maturation, CD8^+^ T lymphocyte infiltration, and CD8^+^/CD4^+^ T cells ratio, dramatically increased the frequency of effector T cells, fewer Tregs, and higher CD8^+^ T /Tregs ratio. (TME)	[122]
BNPs	Decitabine	Breast cancer cells (4T1 cells)	Induced pyroptosis to alleviate the TIME. BNPs + laser induced BMDC maturation in vitro, induced DC maturation, and T cell activation in primary and remote tumor in vivo. (TME)	[123]
SP‐PEG‐PDLLA‐ARV‐825 micelles	ARV‐825	Glioma cells (GL261 cells) and macrophage (RAW264.7 and BMDMs)	Suppressed TAM2 polarization. SP‐PEG‐PDLLA‐ARV‐825 micelles inhibited IRF4 promoter transcription‐mediated TAM2 polarization in BMDMs. (TME‐macrophages)	[124]
LF‐Pan/JQ1 lipos	Panobinostat/JQ1	Colorectal cancer cell (CT‐26) and macrophage (BMDMs)	Induced TAM polarization and tumor glycolysis inhibition to alleviate TIME. LF‐Pan/JQ1 lipos reeducated TAM2 to TAM1 in BMDMs, reduced TAM2 and MDSCs, and upregulated CTL infiltration and NK cells in the CT26 subcutaneous and peritoneal tumor model. (TME‐macrophages)	[125]
Man‐LF‐SHK/JQ1 NPs	JQ1	Colon cancer cells (CT26 cells) and macrophage (BMDMs)	Induced TAM polarization. JQ1 repolarized TAM2 toward TAM2 in BMDMs, triggered promotion of DCs maturation, CD8^+^ T cell infiltration, as well as suppression of TAM2 in vivo. (TME‐macrophages)	[55]
Tra/Man‐Gef/SAHA lipos	SAHA	EGFR‐mutated NSCLC cells (H1975 cells) and macrophage (BMDMs)	Induced TAM polarization. SAHA repolarized TAM2 to TAM1 in BMDMs and EGFR^T790M^‐positive H1975 tumor model. (TME‐macrophage)	[126]
CHI/BMS‐202@lipos	Chidamide	Breast cancer cells (4T1 cells) and DCs (BMDCs)	Induced DC maturation. CHI induced DC maturation in BMDCs, CHI@lipos promoted DC maturation, intratumoral CD4^+^ T cells and CD8^+^ T cell infiltration, and NK cell infiltration in vivo. (TME‐DCs)	[127]

### 3.1 Targeting Tumor Cells for Regulating Apoptosis, Proliferation, Migration, and Therapy Resistance

#### 3.1.1 Monotherapy of Epigenetic Modulator

##### Active Targeting

Vorinostat (suberoylanilide hydroxamic acid, SAHA) was loaded into a pH‐sensitive core composed of poly(lactic‐co‐glycolic acid) (PLGA) and 1,2‐dioleoyloxy‐3‐(trimethylammonium) propane (DOTAP), which was further camouflaged with hybrid membranes generated from red blood cells and metastatic lung cancer cells to construct a novel biomimetic nanovehicle (HRM/PLGA/DOTAP/SAHA NPs). HRM/PLGA/DOTAP/SAHA NPs could target tumors; after hybrid membrane‐mediated homologous targeting, SAHA was released from the nanoparticles in response to intratumoral pH, and then inhibited metastatic lung cancer by restoring H3 acetylation‐driven apoptosis.^[^ ^94^ ^]^ HA‐VRS‐SLNs were prepared by modifying SAHA‐loaded solid lipid nanoparticles (SLNs) with hyaluronic acid (HA) via charge interaction to specifically target tumor cells (e.g., NSCLC cells, SCC cells, and breast cancer cells) overexpressing the CD44 receptor.^[^ ^95^ ^]^

##### Passive Targeting

The intracellular accumulation of the demethylated cytidine analog 5‐AZA is limited by specific membrane nucleoside transporters and instability under physiological conditions. The delivery of DNA demethylation agents based on nanocarriers is a potential development strategy. PLGA‐poly(ethylene glycol) (PEG) diblock conjugated 5‐AZA nanomicelles (AZA‐PLGA‐PEG) were designed, with the characteristics of excellent stability, enhanced intratumor release, high bioavailability, and enhanced anti‐breast cancer cell proliferation.^[^ ^96^ ^]^ Euchromatic histone‐lysine N‐methyltransferase 2 (EHMT2) predominantly transfers methyl from SAM to H3K9 to induce H3K9me1 and H3K9me2; and is thus, considered a potential therapeutic target for hepatocellular carcinoma (HCC). Nanodiamonds (NDs) physically adsorbed with UNC0646, a selective inhibitor of EHMT2, were called complexes (ND‐UNC0646), with good dispersibility and release in response to the acidic tumor environment, thereby showing enhanced HCC treatment efficiency by inhibiting H3K9 methylation.^[^ ^97^ ^]^ The SAHA‐Zn^2+^‐5‐Aza conjugates could self‐assemble into nanofibers with superior antitumor efficiency. Intravenous administration of nanofibers was passively targeted to tumor tissue; SAHA and AZA were released from nanofibers in response to intracellular acid environment and glutathione (GSH); and then, there was synergistic epigenetic reprogramming of cancer cells to induce proapoptosis and antiproliferation of gastric cancer cells.^[^ ^98^ ^]^

##### Local Delivery

HDACi (e.g., panobinostat) with terminally ionizable moieties was loaded into *β*‐cyclodextrin‐poly (*β*‐amino ester) networks (CDN) through the interaction of ions and hydrophobic to self‐assemble into nanoparticles. PEGylated CDN NPs loaded with panobinostat had the characteristics of high drug loading and sustained drug release, and effectively enhanced the concentration of panobinostat in brain tumors via intratumoral convection‐enhanced delivery.^[^ ^91a^ ^]^ To overcome the bladder permeability barrier, belinostat‐loaded PLGA nanoparticles (Bel‐PGON NPs) equipped with surface‐functionalized poly(guanidinium oxanorbornene) (PGON), a positively charged guanidinium group‐based cationic polymer, were prepared. Localized injection of nanoparticles into bladder tumor‐bearing mice via intravesical delivery could effectively adhere to the bladder urothelium and internalize into urothelial and bladder cancer cells; and then, the nanoparticles effectively suppressed bladder tumor growth by increasing the hyperacetylation of histone H4.^[^ ^99^ ^]^

#### 3.1.2 Combination of Epigenetic Therapy and Chemotherapy

Dysregulated hypermethylation and epigenetic silencing of the gene were also associated with drug resistance to chemotherapy. The effectiveness of chemotherapy drugs is usually limited by a narrow therapeutic index^[^ ^100^ ^]^ and resistance,^[^ ^101^ ^]^ which can be overcome by the cooperation of epigenetic drugs.

##### Active Targeting

Cancer cell membrane protein‐disguised liposomes (CCMP‐DOX/SAHA lipos) effectively codelivered DOX and SAHA to tumors through homologous targeting; and thus, facilitated the suppression of non‐small‐cell lung cancer (NSCLC) cells by increasing acetylated histone H3.^[^ ^102^ ^]^ Overexpression of multidrug resistance (MDR) led to poor chemotherapy response and tumor recurrence. General control non‐repressed 5 (GCN5), a histone acetyltransferase, could epigenetically regulate MDR1 expression by enhancing histone acetylation of the MDR1 gene. Mesoporous silica nanoparticles (MSNs‐SS‐pyridine) loaded with DOX were then wrapped with PEI‐*β*‐CD gatekeepers through host–guest interactions (termed DOX@PMSNs). Subsequently, anionic GCN5 siRNA (siGCN5) electrostatically bound to DOX@PMSNs to construct a DOX/siGCN5 codelivering system with hyaluronic acid (HA) modification (DOX/siGCN5@HPMSNs). DOX/siGCN5@HPMSNs could target drug‐resistant breast cancer cells; after CD44‐mediated endocytosis, the released siGCN5 epigenetically abolished P‐glycoprotein (P‐gp) to enhance the intracellular DOX concentration in response to intratumoral pH/GSH; thus, reversing cancer drug resistance and markedly suppressing tumor growth.^[^ ^101^ ^]^

##### Passive Targeting

SAHA‐induced histone hyperacetylation blocked histone–DNA interactions, making DNA more accessible to cisplatin; therefore, SAHA effectively reversed the drug resistance of cisplatin (CDDP) in cancer treatment by increasing strand cross‐links formed by the binding of CDDP and DNA, decreasing intracellular GSH concentrations, and downregulating anti‐apoptotic protein BCL‐2 and multidrug resistance‐related protein (MRP). SAHA‐CDDP‐coupled supramolecular conjugates self‐assembled into nanomicelles, which preferentially accumulated in tumor sites through the EPR effect and further showed remarkable therapeutic efficacy on cisplatin‐resistant NSCLC tumors.^[^ ^103^ ^]^ The low sensitivity of most patients with advanced gastric cancer to 5‐fluorouridine (5‐FU) can be attributed to the hypermethylation of transcription factor AP‐2 epsilon (TFAP2E). The gelatinase‐stimulated‐5‐FU/DAC NPs (5‐FU/DAC‐PEG‐pep‐PCL NPs) were prepared by loading epigenetic drugs (decitabine) and chemotherapy drugs (5‐FU) onto the gelatinase‐stimulated poly (ethylene glycol)‐peptide‐poly (*ε*‐caprolactone) (PEG‐peptide‐PCL) copolymer. The nanoparticles could release drugs in response to the overexpression of matrix metalloproteinases (MMP2/9) and collagenase IV in tumors and restore the expression of TFAP2E through DNA demethylation to resensitize gastric cancer cells to chemotherapy drugs.^[^ ^104^ ^]^

#### 3.1.3 Combination of Epigenetic Therapy and Radiotherapy

##### Passive Targeting

HDACi prevents the radiotherapy‐induced repair of DNA double‐strands (DBS), leading to radiosensitization. PLGA‐lecithin‐PEG NPs were developed by using PLGA loaded with vorinostat or quisinostat. The nanoparticles prolonged the formation of phosphorylated histone H2AX (*γ*‐H2AX), which is a marker of DSB breaks, increased the exposure of radiation‐induced DNA damage; and thus, had the potential to synergistically improve radiotherapy in prostate and colorectal cancers.^[^ ^105^ ^]^ In addition, reactive oxygen species (ROS) produced by radiotherapy can induce DNA damage for tumor killing; therefore, tumor hypoxia may lead to insensitivity to radiotherapy. It has been reported that catalase (CAT) effectively decomposes overexpressed H_2_O_2_ in tumor tissue into oxygen to relieve tumor hypoxia, while HDACi induces histone hyperacetylation to impede the affinity of histones for DNA; thus, consequently triggering radiosensitization. PLGA nanoparticles encapsulated with catalase and SAHA (CAT‐SAHA@PLGA) overcame radiation resistance by synergistically alleviating the hypoxic microenvironment and remodeling chromatin into a loose DNA structure.^[^ ^106^ ^]^ Pt^IV^ could bind with DNA to form platinum‐DNA, thereby making DNA more susceptible to radiotherapy‐generated ionizing radiation. Concomitantly, SAHA could prevent the radiotherapy‐induced repair of DNA through the accumulation of *γ*‐H2AX foci and aggravate DNA damage through the production of ROS. A DNA dual‐targeting nano delivery system (TAT‐Pt/SAHA‐PLGA NPs) for DNA damage aggravation and DNA repair suppression was developed. Briefly, PLGA NPs loaded with the cisplatin prodrug (Pt^IV^) and SAHA were modified with a cell‐penetrating peptide derived from the transactivator of transcription of HIV‐1 for spatial‐temporal codelivery of drugs into tumor tissue; Pt^IV^ and SAHA were released from the nanoparticles in response to the acidic tumoral microenvironment; thus, improving the therapeutic effect of radiotherapy. In addition, nanoplatform‐mediated fluorescence and magnetic resonance imaging realized imaging‐guided accurate radiotherapy and remarkably enhanced therapeutic outcomes.^[^ ^107^ ^]^

#### 3.1.4 Combination of Epigenetic Therapy and Molecularly Targeted Therapy

##### Active Targeting

Gefitinib (Gef), a tyrosine kinase inhibitor (TKI) associated with anti‐epidermal growth factor receptor (EGFR) therapy, has been approved as the first‐line therapy for EGFR‐mutated NSCLC. However, the development of drug resistance has led to molecular‐targeted therapy failure. HA‐PBLG‐Gef/SAHA NPs prepared by hyaluronan‐b‐poly(*γ*‐benzyl‐l‐glutamate) copolymer self‐assembly were internalized by tumor cells through CD44‐mediated endocytosis; the release of Gef and SAHA after intrapulmonary administration synergistically inhibited NSCLC progression.^[^ ^108^ ^]^

##### Passive Targeting

Super‐enhancers (SEs) composed of a large cluster of enhancers with transcriptional activity typically show the enrichment of H3K27ac and oncogenic transcription factors (TFs). BRD4 and cyclin‐dependent kinase 7 (CDK7) are considered important components of SEs and positively regulate SE‐mediated transcription. BRD4 can efficiently mediate chromatin remodeling and transcriptional activation, while CDK7 (a subunit of TFIIH), can promote the initiation and extension of transcription by phosphorylating RNA Pol II. The hydrophobic l‐phenylalanine‐poly(ester amide) nanoparticles (JQ1/THZ1@8P4 NPs) could be loaded with JQ1 (a BRD4 inhibitor) and THZ1 (a CDK7 inhibitor) to target tumor sites. JQ1/THZ1@8P4 NPs achieved effective inhibition of gemcitabine‐resistant PDAC by restraining tumor cell proliferation, migration, and invasion through the inhibition of SE‐associated TFs.^[^ ^109^ ^]^ Zein nanoparticles (ZNP/SB NPs) loaded with SAHA and bortezomib (Bor, a proteasomal inhibitor) were successfully prepared via the phase separation method. The ZNP/VB NPs promoted the accumulation of ubiquitination proteins based on SAHA‐enhanced protein unfolding and Bor‐prevented unfolded protein degradation to drive ER stress‐mediated apoptosis; and thus, treated metastatic prostate cancer by passively targeting the tumor site.^[^ ^110^ ^]^

#### 3.1.5 Combination of Epigenetic Therapy and Other Therapies

##### Active Targeting

The treatment strategy of epigenetic modulators and PD‐1/PD‐L1 blockade may be a novel cancer treatment approach. The disulfide‐containing poly (*β*‐amino ester) (ssPBAE) nanocore with surface‐modified iRGD peptide conjugated propylene glycol alginate sodium sulfate (iRGD‐PSS) could be loaded with JQ1 and oridonin (ORI, bioactive diterpenoid derived from traditional Chinese medicine herb) to target the tumor. The iRGD‐PSS@PBAE@JQ1/ORI NPs effectively enhanced tumor targeting due to the tumor‐overexpressed *α*v*β*3 integrin receptors; JQ1 and ORI were released from nanoparticles in response to intracellular pH/GSH and achieved remarkable synergistic anti‐breast cancer efficiency by reversing PD‐L1‐mediated immune tolerance, increasing intracellular ROS production, and inhibiting lactic acid secretion.^[^ ^111^ ^]^ Malignant tumors are usually associated with overexpression of HDAC. A novel nanoplatform (M1‐EM‐SUCS NPs) was established in which M1 macrophage‐derived exosome membranes camouflaged mesoporous silica‐modified lanthanide‐doped upconversion nanoparticles (UCs) loaded with SAHA. The nanoparticles could be internalized by tumor cells through the interaction between integrin *α*4*β*1 on the M1 macrophage‐derived exosome and ICAM1; thus, achieving spatiotemporal‐resolved delivery and improving Lewis lung cancer therapy by promoting apoptosis driven by histone acetylation including H3K27ac and H3K9ac. In addition, M1‐EM‐SUCS NPs could be tracked by PA imaging with the aid of UCs and laser irradiation; thus, achieving real‐time diagnosis.^[^ ^112^ ^]^

##### Passive Targeting

Triple‐negative breast cancers (TNBCs) lacking the expression of estrogen receptor *α* (ER*α*), progesterone receptor (PR), and human epidermal growth factor 2 (HER2) have poor sensitivity to tamoxifen (a selective estrogen receptor regulator). SAHA can reactivate HDAC‐mediated ER*α* gene silencing and repair the response of tamoxifen to TNBCs, indicating that the combination of HDACi and tamoxifen may provide a novel therapy for TNBCs. A redox‐responsive SAHA‐based prodrug polymer (POEG‐co‐PVDSAHA) was prepared by incorporating SAHA into the polymer backbone with a disulfide linkage. POEG‐co‐PVDSAHA, as an amphiphilic polymer, self‐assembled to form tamoxifen‐loaded micelles (POEG‐co‐PVDSAHA/TAM) that resensitized TNBCs to tamoxifen therapy and significantly improved the anti‐breast cancer effect.^[^ ^113^ ^]^

### 3.2 Targeting Tumor Cells for Tumor Immune Microenvironment Modulation

#### 3.2.1 Monotherapy of Epigenetic Modulator

##### Active Targeting

The core nanoparticles loaded with ORY‐1001 (a selective inhibitor of LSD1) were developed by cross‐linking bovine serum albumin (BSA) with NHS‐SS‐NHS; and then, functionalized with programmed cell death protein 1 (PD1)‐expressing T lymphocyte membrane and GSH‐activated pore‐forming activity‐caged macrolittin 70 (cM70). The BSA‐ORY@TM/cM70 NPs could target tumor cells; after PD‐L1‐mediated endocytosis, ORY‐1001 was released from nanoparticles with the aid of M70 and then replenished intratumoral IFNs and tumor‐infiltrating lymphocytes through LSD inhibition‐induced H3K4me1 and H3K4me2 accumulation; thus, effectively inhibiting xenograft tumor growth, including TNBC, melanoma, and colon cancer.^[^ ^114^ ^]^

#### 3.2.2 Combination of Epigenetic Therapy and Chemotherapy

##### Passive Targeting

The prodrug‐based coassembled nanogels were prepared, by which LAQ824 (LAQ, an HDACi) and DOX were crosslinked by 4‐nitrophenyl chloroformate‐SS‐4‐nitrophenyl chloroformate (DBHD); thus, self‐assembling into GSH‐sensitive nanogels (LAQ/DOX‐DBHD nanogels). The released LAQ and DOX from LAQ/DOX‐DBHD nanogels collaboratively drove pyroptosis‐induced immune activation; and thus, significantly exerted remarkable treatment efficiency in breast cancer and SCC. Furthermore, LAQ/DOX‐DBHD nanogels could significantly improve anti‐PD‐1 therapy.^[^ ^115^ ^]^

#### 3.2.3 Combination of Epigenetic Therapy and Molecularly Targeted Therapy

##### Active Targeting

Indoleamine 2,3‐dioxygenase (IDO) can metabolize tryptophan (Trp) to kynurenine (Kyn), which leads to the inhibition of T‐cell infiltration and the recruitment of Tregs. An ultrasmall Cu_2−_ *_x_* Se nanoparticles functionalized with a layer of cancer cell membrane were developed to deliver indoximod (IND, an IDO‐1 inhibitor) and JQ1 (a PD‐L1 inhibitor). The biomimetic CS‐I/J@CM NPs could pass through the BBB with the assistance of noninvasive focused ultrasound and enhance accumulation in the tumor through membrane‐mediated homologous targeting after intravenous injection. CS‐I/J@CM NPs greatly boosted the antitumor immune response by remodeling the tumor immunosuppressive microenvironment (TIME) of glioblastoma through various mechanisms, including hypoxia alleviation‐induced TAM repolarization, IND‐mediated inhibition of Treg cells, and JQ1‐restricted PD‐L1 expression. In addition, the combination of CS‐I/J@CM NPs and NIR II irradiation showed excellent treatment efficacy on glioblastoma by remodeling the TIME, inducing ICD‐activated immunity, and preventing tumor recurrence through the enhancement of immune memory.^[^ ^116^ ^]^ The PLGA NPs loaded with brefeldin A (BFA, a subcellular transport inhibitor) and JQ1 were camouflaged with a homologous membrane (HCC‐PLGA‐BFA/JQ1 NPs), which prevented lysosome entrapment via caveolae‐related endocytosis and gradually accumulated in the endoplasmic reticulum (ER) and Golgi apparatus with the aid of the SNARE protein‐associated trafficking pathway and BFA‐induced coat protein type I (COPI) vesicle transport inhibition; thus, exerting priority and long‐term ER retention. The BFA released from nanoparticles could promote ER stress‐associated ICD and CD8^+^ T‐cell recruitment, and combined with JQ1 overcame ICD‐induced PD‐L1 enrichment; thus, synergistically improving the anti‐melanoma therapy efficiency and boosting long‐term antitumor immunity in a CD8^+^ T‐cell‐dependent manner.^[^ ^117^ ^]^

##### Passive Targeting

Tumor‐infiltrating CTL‐secreted IFN‐*γ* can induce adaptive immune resistance by activating IDO‐1 and PD‐L1. IDO‐1 can activate Tregs and consume tumor‐infiltrating CTLs by metabolizing Trp into Kyn, while PD‐L1, which is overexpressed on tumor cells, can trigger CTL depletion through interaction with PD‐1 expressed on T cells. JQ1, a BRD4 inhibitor, can eliminate the IFN‐*γ*‐induced PD‐L1 expression. NLG919 can inhibit IDO‐1‐mediated consumption of Trp to reshape the antitumor immune response. Bispecific prodrug nanoparticles (PHPNJ NPs) were first prepared by self‐assembly of disulfide‐linked NLG919 and JQ1 (NLG919‐SS‐JQ1, NJ), and then, coated with a photosensitizer‐modified (pyropheophorbide‐a, PPa) and tumor acid‐responsive diblock copolymer PPa‐conjugated mPEG_113_‐b‐P(HMA50‐r‐HEMA5) (PHP) for passive targeted delivery to tumor tissue. The prodrug PHPNJ NPs achieved tumor‐specific delivery of NJ due to acid‐responsive mPEG_113_‐b‐P(HMA_50_‐r‐HEMA_5_) shell cracking; after glutathione‐triggered cleavage of the disulfide bond, JQ1 and NLG919 were released from the NJ, and then yielded the antitumor immune response by preventing two immune escape pathways, including PD‐L1‐mediated CTL exhaustion and IDO‐1‐triggered Trp consumption. Apart from the combination of epigenetic agents and molecularly targeted drugs, the PHPNJ NP‐activated antitumor immune response symphysis with photodynamic therapy (PDT)‐triggered immunogenic cell death (ICD) of tumor cells showed significant anti‐colon and breast cancer effects.^[^ ^118^ ^]^ DNMTi‐induced PD‐L1 expression resensitized tumors to PD‐L1 checkpoint blockade therapy. However, adenosine triphosphate (ATP) in tumors was hydrolyzed into immunosuppressive adenosine (ADO) by DNMTi‐reactivated CD73, which greatly hindered CTL infiltration into tumors and elicited TIME. The *α*, *β*‐methylene adenosine 5’ diphosphate (AMPCP), a CD73 inhibitor, directly triggered DNMTi‐induced gasdermin D (GSDMD) cleavage through ATP‐activated caspase‐1, and subsequently, the N‐terminal fragment of GSDMD formed membrane pores to drive pyroptosis. In addition, the release of inflammatory molecules through the GSDMD‐N pore could facilitate the recruitment of immune cells in the TME. GSH‐responsive prodrug nanomicelles (AOZNs) loaded with DNMTi (*γ*‐oryzanol, Orz) and AMPCP were prepared by crosslinking disulfide bonds (DBHD) with free drugs with hydroxyl groups. After passively accumulating AOZNs in tumor tissue through the EPR effect, Orz and AMPCP were released from nanomicelles in response to GSH in the TME, and then, synergistically induced GSDMD‐mediated pyroptosis in tumor cells and alleviated the TIME through the promotion of T‐cell infiltration and DC maturation and the suppression of immunosuppressive MDSC accumulation and TAM2 polarization. In addition, AOZNs induced a strong antitumor immune response in the B16F10 model, resensitized tumors to anti‐PD‐L1 therapy through the enhancement of PD‐L1 expression, and efficiently regressed tumor growth.^[^ ^119^ ^]^

#### 3.2.4 Combination of Epigenetic Therapy and Photothermal Therapy

##### Passive Targeting

The second near‐infrared (NIR‐II) photothermal‐mediated liposomes (CuS/CpG/JQ1 lipos) encapsulating copper sulfide (CuS, an NIR‐II photothermal agent), cytosine–phosphor–guanine oligodeoxynucleotides (CpG ODNs, a TLR‐9 agonist), and JQ1 (PD‐L1 inhibitors) were constructed, achieving NIR‐II laser‐induced ICD as well as the release of immune agents (JQ1 and CpG ODNs). CuS/CpG/JQ1 lipos facilitated antitumor immune responses, including the enhancement of DC maturation and CTL infiltration, by combining the induction of ICD, activation of toll‐like receptor‐9 (TLR‐9), and suppression of PD‐L1. In addition, CuS/CpG/JQ1 lipo‐mediated photothermal‐synergized immunotherapy radically inhibited the growth of primary and distant PDAC and breast cancer, and effectively prevented pulmonary metastasis.^[^ ^120^ ^]^

##### Local Delivery

The polydopamine nanoparticles loaded with JQ1 (PDMN‐JQ1 NPs) were prepared through *π*–*π* stacking interactions. Intratumoral administration of PDMN‐JQ1 NPs synthesized with photothermal therapy not only promoted apoptosis and PD‐L1 expression through the suppression of the BRD4‐c‐Myc axis but also boosted the antitumor immune response and immune memory effect through the activation of T lymphocytes and central memory T cells, further effectively regressing the growth and recurrence of primary and distal TNBCs.^[^ ^121^ ^]^

#### 3.2.5 Combination of Epigenetic Therapy and Photodynamic Therapy

##### Active Targeting

A supramolecular prodrug nanoplatform (HA‐CD‐PPa/JQ1 NP) was prepared through the host–guest interaction between cyclodextrin‐grafted hyaluronic acid (HA‐CD) and adamantine conjugated heterodimers (AD‐SS‐JQ1 and AD‐SS‐PPa) to target tumor cells overexpressing CD44; after CD44‐mediated endocytosis, pyropheophorbide a (PPa) and JQ1 were released from the nanoplatform in response to intracellular GSH. PPa‐mediated PDT enhanced ICD‐induced DC maturation and CTL tumor infiltration. Simultaneously, JQ1 counteracted PDT‐mediated immune evasion by blocking c‐Myc‐initiated glycolysis and PD‐L1‐induced immunosuppression. HA‐CD‐PPa/JQ1 NP‐mediated photoimmunotherapy effectively suppressed the growth and metastasis of pancreatic cancer.^[^ ^122^ ^]^ Pyroptosis, an inflammatory programmed cell death process, can be initiated by the caspase‐cleaved gasdermin family and the release of pro‐inflammatory intracellular content, which can alleviate immunosuppression in tumors. Recently, it has been reported that demethylation of low‐dose decitabine could promote gasdemin‐mediated programmed necrosis. Biomimetic nanoparticles (BNPs) formed by the fusion of breast cancer membrane onto the PLGA copolymer core were loaded with indocyanine green (ICG) and decitabine, which synergistically enhanced the pyroptosis via the Ca2^+^‐dependent caspase‐3 induced by photosensitizer and the caspase‐3‐cleaved GSDMD induced by DNA demethylation agent (decitabine, DAC). BNPs were highly accumulated in the tumor through photoactivated drug release and cancer cell membrane‐mediated tumor homing, and mediated cell pyroptosis triggered a systemic antitumor immune response; thus, preventing the primary tumor and metastasis of breast cancer.^[^ ^123^ ^]^

### 3.3 Targeting Antigen‐Presenting Cells for Tumor Immune Microenvironment Modulation

#### 3.3.1 Monotherapy of Epigenetic Modulator

##### Active Targeting

BRD4 is positively correlated with massively infiltrated glioma macrophages, and high expression of the BRD4 gene and TAM2 is linked with poor prognosis in glioma patients. BRD4 promoted TAM2 polarization through the inhibition of IRF4 promoter transcription, which can be inhibited by ARV‐825 (a BRD4 inhibitor) based on proteolytic targeting chimera technology. The substance P (SP) peptide can specifically bind to the neuroprotein 1 receptor (NK‐1R), which is overexpressed in glioma cells and endothelial cells. The SP peptide‐functionalized PEG‐poly(d,l‐lactic acid)(SP‐PEG‐PDLLA) and methoxy PEG‐poly(d,l‐lactic acid) (mPEG‐PDLLA) could form a complex micelle (SP‐PEG‐PDLLA‐ARV‐825) for loading ARV‐825 to penetrate the BBB and target brain tumors. Intravenous administration of the micelle suppressed glioma growth through anti‐angiogenesis, pro‐apoptosis, and IRF4 promoter transcription‐mediated TAM2 polarization inhibition.^[^ ^124^ ^]^ Lactoferrinized liposomes (LF‐Pan/JQ1 lipos) codeliver panobinostat (Pan, an HDACi) and JQ1 to cancer cells and TAMs overexpressing LRP‐1 and albumin‐binding proteins (SPARC). LF‐Pan/JQ1 lipos could be considered a dual‐target therapy to boost the tumor immune response, repolarize TAMs, recruit tumor effector CD8^+^ T‐cell infiltration, and suppress angiogenesis and glucose metabolism, thereby remodeling the tumor immune microenvironment. LF‐Pan/JQ1 lipos suppressed colorectal tumor growth and metastasis; furthermore, combined with anti‐PD‐L1 therapy effectively inhibited tumor recurrence.^[^ ^125^ ^]^

#### 3.3.2 Combination of Epigenetic Therapy and Other Therapies

##### Active Targeting

Lactoferrin can specifically bind with low‐density lipoprotein receptor‐related protein 1 (LRP‐1) overexpressed on the surface of cancer cells and macrophages. The mannosylated lactoferrin nanoparticles (Man‐LF‐SHK/JQ1 NPs) were prepared for delivering shikonin (a naphthoquinone pigment isolated from the traditional Chinese herb Zicao) and JQ1 (a PD‐1 blockage agent) to cancer cells and TAMs via mannose receptor (MR) and LRP1. The Man‐LF‐SHK/JQ1 NPs had the antitumor ability to reshape the tumor immune microenvironment via the repression of glucose metabolism, repolarization of TAMs, initiation of ICD, and JQ1‐mediated PD‐1 blockage.^[^ ^55^ ^]^ SAHA‐mediated inhibition of HDAC2 may be associated with the repolarization of TAM2 to TAM1, which could overcome drug resistance in EGFR‐TKI therapy. The Tra/Man‐Gef/SAHA liposomes were developed by dual camouflaging with mannose (Man) and trastuzumab (Tra) for dual targeting of both TAM2 and HER2‐positive NSCLC cells to overcome EGFR^T790M^‐associated drug resistance. The liposomes had the antitumor ability to reprogram the protumor TAM2 toward antitumor TAM1 and modulate the ROS/methionine sulfoxide reductase A (MsrA)/EGFR^T790M^ transduction axis; thus resensitizing EGFR^T790M^‐associated Gef‐resistant NSCLC therapy.^[^ ^126^ ^]^

##### Passive Targeting

TNBCs, as a “cold” tumor, severely limited the efficiency of ICB therapy, while chidamide (CHI), a selective HDACi, enhanced the antitumor immunoreactivity of ICB treatment by inducing ICD, including calreticulin (CRT) eversion, high mobility group box 1 (HMGB1) release, and adenosine triphosphate (ATP) secretion. A liposome system (CHI/BMS‐202@lipF) was constructed by encapsulating the Bristol–Myers Squibb (BMS‐202, PD‐L1 inhibitor) and CHI‐F127 complex, consisting of Pluronic F127 and CHI, into liposomes via reverse evaporation. Furthermore, CHI/BMS‐202@lipF could passively target the tumor site and facilitate the release of the drug continuously after intravenous administration; thus, synergistically enhancing T‐cell‐mediated antitumor immunity and effectively inhibiting the growth and metastasis of breast cancer.^[^ ^127^ ^]^

### 3.4 Overview of Targeting Strategy

After systemic administration, nanoparticles are easily captured by the reticulo‐endothelial system (RES); the abnormal tumor vasculature, high interstitial fluid pressure, and dense extracellular matrix in the tumor microenvironment limit the extravasation and diffusion of nanoparticles.^[^ ^128^ ^]^ The development of smart nanoparticulate formulations was intended to overcome several physical and physiological barriers, and then, cargos could be internalized by targeting tumor cells. Several nanomedicines can target the TME via EPR‐mediated passive targeting, active targeting which relies on receptor‐mediated targeting, and TME (e.g., hypoxia, low pH, enzyme, and redox species)–triggered targeting.^[^ ^129^ ^]^

The heterogeneity within the TME holds great challenges for delivery efficiency. Inspired by this, active targeting nanotechnology has been proposed to selectively deliver cargo to tumor cells. Ligand‐grafted nanomedicines with preferable tumor accumulation such as trastuzumab (Tra)‐mediated NSCLC (HER‐2 positive) targeting and substance P peptide‐mediated glioma targeting, have been developed to facilitate specific cancer delivery and increase therapeutic efficacy.^[^ ^124^, ^126^ ^]^ Overexpression of *α*v*β*3 integrin receptors, CD44, and mannose receptors in diverse tumor types (e.g., colorectal cancer, breast cancer, and NSCLC) also provides a potential target for iRGD, HA, or mannose‐modified nanoparticulate formulations.^[^ ^55^, ^95^, ^101^, ^108^, ^111^ ^]^ Furthermore, biomimetic nanoparticles including cell membrane‐camouflaged nanoparticles (e.g., homologous cancer cell membranes and PD1‐expressing T lymphocyte membrane),^[^ ^94^, ^102^, ^114^, ^116^, ^117^, ^123^ ^]^ exosome‐disguised nanoparticles (e.g., M1‐derived exosome),^[^ ^112^ ^]^ albumin nanoparticles,^[^ ^114^ ^]^ and lactoferrin nanoparticles,^[^ ^55^ ^]^ served as novel nanotechnology for targeting tumor cells and regulating the TME in the treatment of breast cancer, colorectal cancer, glioma, melanoma, and lung cancer.

These smart nanoparticulate formulations discussed in the current review include liposomes, solid lipid nanoparticles, polymeric nanoparticles, polymeric micelles, polymer–drug conjugate nanoparticles, prodrug nanoparticles, and inorganic nanoparticles, with an emphasis on polymeric nanoparticles. Polymeric nanoparticles are characterized by increased drug targeting, minimized systemic side effects, as well as enhanced bioavailability. Various polymers (e.g., polydopamine, poly (*β*‐amino ester), and poly(ester amide)) and copolymers (e.g., PLGA, PEG‐PDLLA, PEG‐PCL, POEG‐co‐PVD, mPEG113‐b‐P(HMA50‐r‐HEMA5)) are recommended as superior nanocarriers because of their high biocompatibility and biodegradability, as well as being non‐toxic.^[^ ^130^ ^]^ To overcome en‐route barriers (e.g., RES, BBB, and bladder epithelial barrier),^[^ ^131^ ^]^ PLGA NPs have been modified with PEG (e.g., AZA‐PLGA‐PEG, PLGA‐lecithin‐PEG NPs) to escape RES and extend blood circulation.^[^ ^96^, ^105^ ^]^ The substance P peptide‐functionalized PEG‐PDLLA micelle (SP‐PEG‐PDLLA‐ARV‐825) could penetrate the BBB and target brain tumors.^[^ ^124^ ^]^ In addition, the poly(guanidinium oxanorbornene) (PGON)‐equipped PLGA NPs effectively overcome the bladder epithelial barrier.^[^ ^99^ ^]^ As for cellular barriers (cellular uptake limitation and lysosomal degradation),^[^ ^131^ ^]^ nanocarriers such as cell‐penetrating peptide (TAT peptide)‐modified PLGA NPs (TAT‐PEG‐b‐PLGA) increased greater transport and improved cellular uptake of nanoparticles.^[^ ^107^ ^]^ Several targeting ligands such as RGD peptide were introduced to polymeric nanocarriers (ssPBAE), resulting in receptor‐mediated tumor targeting and increased cellular uptake.^[^ ^111^ ^]^ To achieve TME delivery, gelatinase‐cleavable peptide (PEG‐peptide‐PCL), disulfide (POEG‐co‐PVDSAHA), and amides (mPEG113‐b‐P(HMA50‐r‐HEMA5)) were attached to copolymer, respectively, leading to TME (e.g., GSH, pH, and gelatinase)–triggered targeting.^[^ ^104^, ^113^, ^118^ ^]^

## 4 Conclusions, Challenges, and Perspectives

Epigenetic changes have an important influence on cancer progression; therefore, epigenetic modulators have emerged as an attractive treatment for malignant tumors and regulate the sensitivity of tumors to other therapies. Epigenetic reprogramming of tumor cells and immune cells in the TME can regulate tumor immune response and affect the progression of the tumor. Epigenetic modification directly regulates the phenotype and function of immune cells (e.g., TAMs, CAFs, and T cells); thus affecting the progression, metastasis, drug resistance, and immune evasion of tumors. Based on the crosstalk between cancer cells and macrophages, epigenetic regulation of tumor cells can further control the polarization, recruitment, and infiltration of TAMs by modulating pro‐inflammatory cytokine and chemokine pathways. Beside, epigenetic regulation of Th1‐type chemokines secreted by tumor cells may drive chemokine‐mediated effector T‐cell homing. Insight into the interaction between epigenetic modifications and immune and stromal cells in the TME (e.g., TAMs, CAFs, and T lymphocytes) during the process of cancer development will help clarify the antitumor mechanism and rationally design delivery and treatment strategies.

Epigenetic therapies, including inhibitors of DNMT, HDAC, BET, and LSD, have been developed and are available for cancer therapy. However, the poor water solubility, rapid clearance, and poor tissue distribution of epigenetic agents have been disappointing. Epigenetic monotherapies or synergy with other therapies (including chemotherapy, radiotherapy, molecularly targeted therapy, photothermal therapy, photodynamic therapy, photoacoustic imaging, immunotherapy, hormone therapy, and other therapies) show remarkable efficiency in the treatment of solid tumors. One way to implement the combination of epigenetic agents and other antitumor agents is to take advantage of advanced nanotechnology that could provide potential therapeutic strategies for combined application and simultaneous delivery. A growing body of studies have shown that nanomedicine delivery systems had the characteristics of high drug loading, sustained drug release, increased stability, precise targeting, and enhanced drug concentration in the tumor. Consistent with these effects, nanomedicine can be specifically delivered to tumor tissue rather than to normal tissue after systemic administration, which considerably improves the accurate nanomedicine delivery and prevents off‐target toxicity. Furthermore, after delivery to tumor tissue, epigenetic agents and other antitumor agents can be released from the nanovehicles in response to the TME (e.g., pH, GSH, and enzyme) in a spatio‐temporal manner to exert optimal synergetic effects. As emphasized throughout this review, the epigenetic‐based nano delivery strategy promotes apoptosis, impedes proliferation and migration, and overcomes therapy resistance by targeting tumor cells, as well as remodels the tumor immune microenvironment by targeting tumor cells and APCs (e.g., macrophage and DCs). Moreover, natural products and their derivatives have been extensively studied as epigenetic modulators to regulate the tumor microenvironment.^[^ ^132^ ^]^ With the aid of advanced delivery strategies, the combination of natural products with excellent safety index and marketed antitumor drugs may better realize epigenetic‐based nanotechnology for cancer therapy. Collectively, epigenetic‐based nanoplatform can better enhance the anti‐tumor therapeutic efficiency of other therapies by regulating TME (e.g., tumor cells and immune cells). Given therapeutic resistance reversal and TME modulation benefits from epigenetic agent synergy with other anticancer therapies, precision nanomedicine is a promising future for the development of epigenetic‐based therapies.

However, the ‘one size fits all’ approach is not suitable for all patients clinically due to tumor heterogeneity; great efforts should be focused on predictive biomarkers for different patients to realize more effective individualized treatment. Furthermore, as the understanding of the functional mechanisms of different epigenetic regulators is deepened, the targeting of the epigenetic regulator can be further optimized by nanotechnology based on spatio‐temporal approaches. In addition, although TME remodeling may be achieved by regulating tumor cells and APCs, further research is still necessary to develop a nanoplatform that can epigenetically and synergistically regulate stromal cells and other immune cells to improve antitumor treatment efficacy. Nonetheless, together with an extensive understanding of the role of epigenetic modification in the regulation of the TME, the epigenetic‐based nano delivery strategy will eventually enable us to develop the full potential of epigenetic agents in treating tumors.

## Conflict of Interest

Leaf Huang is a consultant for the Samyang Biopharmaceutical company, PDS Biotechnology, and Stemirna Therapeutics. Other authors declare no conflict of interest, financial or otherwise.
